# Resonator nanophotonic standing-wave array trap for single-molecule manipulation and measurement

**DOI:** 10.1038/s41467-021-27709-3

**Published:** 2022-01-10

**Authors:** Fan Ye, James T. Inman, Yifeng Hong, Porter M. Hall, Michelle D. Wang

**Affiliations:** 1 Howard Hughes Medical Institute, Ithaca, NY 14853 USA; 2 Department of Physics & LASSP, Ithaca, NY 14853 USA; 3 Department of Electrical and Computer Engineering, Ithaca, NY 14853 USA; 4 Biophysics Program, Ithaca, NY 14853 USA

**Keywords:** Optical tweezers, Nanophotonics and plasmonics, Nanoscale biophysics, Single-molecule biophysics, Optical manipulation and tweezers

## Abstract

Nanophotonic tweezers represent emerging platforms with significant potential for parallel manipulation and measurements of single biological molecules on-chip. However, trapping force generation represents a substantial obstacle for their broader utility. Here, we present a resonator nanophotonic standing-wave array trap (resonator-nSWAT) that demonstrates significant force enhancement. This platform integrates a critically-coupled resonator design to the nSWAT and incorporates a novel trap reset scheme. The nSWAT can now perform standard single-molecule experiments, including stretching DNA molecules to measure their force-extension relations, unzipping DNA molecules, and disrupting and mapping protein-DNA interactions. These experiments have realized trapping forces on the order of 20 pN while demonstrating base-pair resolution with measurements performed on multiple molecules in parallel. Thus, the resonator-nSWAT platform now meets the benchmarks of a table-top precision optical trapping instrument in terms of force generation and resolution. This represents the first demonstration of a nanophotonic platform for such single-molecule experiments.

## Abstract (web summary)

Applications of nanophotonic tweezers have been limited by the low trapping force. Here, the authors present enhanced force generation in a nanophotonic standing-wave array trap by integrating a critically-coupled resonator design and demonstrate common single-molecule experiments.

## Introduction

Optical trapping is a cornerstone biophysical technique to investigate biological systems^1,2^ and has allowed unprecedented insight into molecular interactions, including measuring the forces, torques, and step sizes of motor proteins^3–6^, examining the kinetics of protein unfolding^7^, probing the biophysical properties of DNA^8–11^ and RNA^12^, and disrupting proteins bound to DNA^13–15^. Despite this, broader accessibility to this technology has been hampered by the requirements of specialized laboratory space and complex table-top equipment, as well as the inherent low throughout of manipulating one molecule at a time.

Nanophotonic trapping (or nanophotonic tweezers) presents a promising solution. Using near-field evanescent waves at the surface of an on-chip structure, these new trapping platforms can be mass-produced, are efficient at trapping particles at high throughput, are more compact than traditional optical tweezers, and are inherently robust against noise. Thus, they open new opportunities for the manipulation and measurement of individual biological molecules on-chip^16,17^.

However, to realize their full potential and enable comprehensive implementation, these platforms must match the performance afforded by precision table-top optical tweezers, characterized by nm spatial resolution, μm-scale manipulation, and tens of pN of applied forces. For instance, the mechanical unzipping of a DNA molecule through a bound protein represents a powerful single-molecule tool for mapping the location of the bound protein^18–21^. This application requires around ~10 pN forces to unzip DNA of thousands of base pairs (bp) (or thousands of nanometers, nm) to locate a bound protein to near nm accuracy and precision. Thus, this type of experiment is essential in optical trapping instruments.

**Fig. 1:**
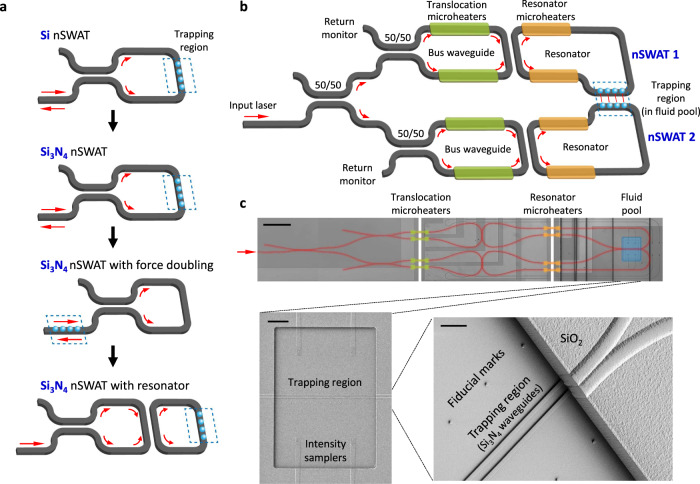
Design and fabrication of a resonator-nSWAT device. **a** Development progression of the nSWAT platform. Gray structures denote waveguides, and red arrows indicate light propagation. **b** Design of the resonator-nSWAT. Two copies of the Si_3_N_4_ nSWAT are powered by the same light source. For each nSWAT, the trapping region resides within the resonators which is tuned by the resonator microheaters, and the trap position is controlled by the translocation microheaters. See main text for a detailed description. **c** Images of a resonator-nSWAT device. Top: Optical image of a device. False coloring is used to highlight specific components: primary waveguides (red), translocation (green) and resonator (yellow) microheaters, and fluid pool (blue). Not highlighted but visible are 8 light intensity samplers, 4 for the two bus waveguides and 4 for the two resonator waveguides. Each light intensity sampler is coupled to the primary waveguide via a 1/99 splitter. For compactness, a vertical break space is used to represent a 3.4-mm region with translocation microheaters and a 3.0-mm region with resonator microheaters. Scale bar is 200 μm. Bottom left: A SEM image of the fluid pool containing waveguides of the trapping region. The scattering gratings of the four light intensity samplers from the two resonators are clearly visible. Scale bar is 20 μm. Bottom right: A zoomed-in and tilted view of a SEM image of the fluid pool, showing high quality Si_3_N_4_ waveguides at the trapping region within the fluid pool. Scale bar is 5 μm. SEM images were taken for every resonator-nSWAT device to verify the fabrication. This SEM image is representative of a typical device. We have fabricated more than 30 nSWAT devices.

Although this performance expectation has presented substantial challenges for previous nanophotonic structures^22–31^, a promising platform is the recent development of the nanophotonic standing-wave array trap (nSWAT)^32^ (Fig. 1a; Supplementary Movie 1) that is capable of precision position control and flexible relocation of a trap array. In this platform, an evanescent standing wave is formed via two waves counter-propagating in a nanophotonic waveguide, creating an array of trapping centers at the anti-nodes of the evanescent standing wave. Using an on-chip microheater, the entire trap array may then be precisely relocated via thermo-optic modulation of the phase difference between the two waves. Unlike conventional optical traps, the nSWAT creates an array of traps by recycling the same light without proportionally increasing the light power.

Since the inception of the nSWAT^32^, significant effort has been devoted towards meeting the expectations for a precision on-chip optical trapping platform (Fig. 1a). The first generation nSWAT, which was based on Si waveguides, demonstrated nm-precision control for relocating trapped particles over microns of distance at a rate faster than kHz^32^. Although it established a significant foundation, Si waveguides have strong non-linear absorption of light, limiting the power deliverable to the trapping region. Furthermore, Si is only transparent to light at a wavelength at which aqueous solution absorbs light strongly, leading to significant sample heating that is not desirable for biological experiments. Thus, the second generation nSWAT was instead based on Si_3_N_4_ waveguides^33^. Si_3_N_4_ is transparent to a 1064-nm trapping laser, greatly reducing light absorption by both the waveguides and the aqueous solution. Subsequently, a variation of the Si_3_N_4_ nSWAT design has further allowed the doubling of light at the trapping region^34^, and the use of high index trapping particles has substantially enhanced trap stiffness^35^.

Despite these advances, inherent to all previous nSWAT designs was the inevitable property that light passing through the trapping region is ultimately returned to the waveguide input by retracing the input light path (Supplementary Movie 2). This return light may cause back-reflection to the laser source and induce waveguide burning near the input coupling region, limiting these devices to low-force applications.

In this work, we present the next-generation nSWAT, which can generate sufficient force for precision single-molecule measurements. This design incorporates a critically-coupled design which significantly minimizes the return light, while permitting local light enhancement at the trapping region. Furthermore, we have incorporated a novel microheater modulation scheme to enable sustained high force generation over a long manipulation distance. These two features have permitted significant trapping force enhancement. These enhancements have enabled standard optical trapping applications, including stretching DNA molecules to measure their force-extension relations, unzipping DNA molecules, and disrupting and mapping protein–DNA interactions.

## Results

### Resonator-nSWAT design and fabrication

The current nSWAT devices were designed and fabricated by integrating Si_3_N_4_ nanophotonic resonators, 3-dimensional (3D) microelectronic structures, and a microfluidic channel all on-chip, in a sophisticated, multi-layered configuration (Fig. 1b, c; “Methods”; Supplementary Figs. 1–3; Supplementary Methods 1–4). An external 1064 nm laser is coupled fiber-optically to each device through a tapered nanophotonic waveguide. The light is then split via a 50/50 beam splitter into two paths, which are directed to two separate nSWATs that work in conjunction in an experiment. Stable coupling is maintained via a suspended sample holder design (Supplementary Fig. 4; Supplementary Method 5). The single-molecule sample is sent into the nSWAT flow chamber (“Methods”) through a gravity flow cell system (Supplementary Method 6). In each nSWAT, the light passes through another 50/50 beam splitter that creates counter-propagating waves to form a standing wave in a loop which also serves as the bus waveguide for coupling to an adjacent resonator. Thus a standing wave is also formed in the resonator, where the trapping region resides, and whose resonance is tuned via on-chip ‘resonator heaters’ located over the resonator. To translocate the array trap, ‘translocation microheaters’ located over the bus waveguide modulate the phase of the light to control the position of the standing wave in the resonator (Supplementary Movie 3). Thus, the two nSWATs are independently controlled and tuned.

### Critical coupling and resonance tuning

**Fig. 2:**
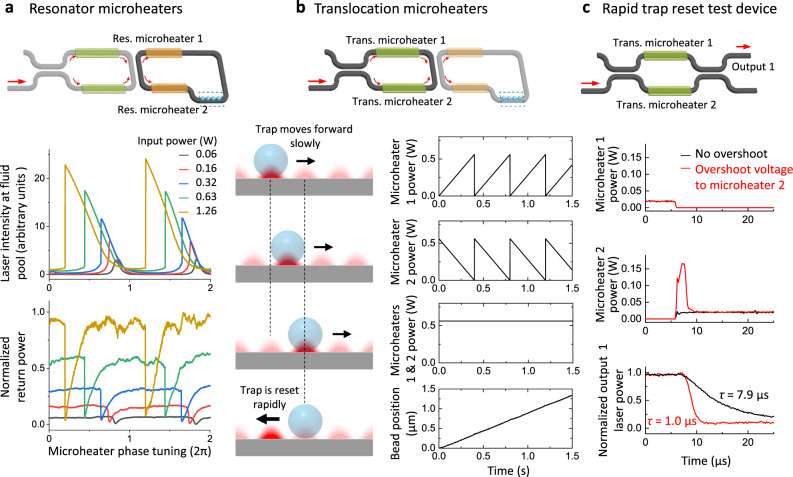
Control and characterization of the resonators and microheaters. **a** Resonator response curves. For each resonator, its phase is concurrently modulated by two identical resonator microheaters (top panel), which sandwich the trapping region and operate in a parallel mode. This design minimizes phase changes of the standing wave in the trapping region that might be introduced during resonator tuning. The laser power indicated refers to the power at the output of the fiber-optic coupler prior to coupling to the nSWAT. At a high laser power, the phase response of the resonator near the resonance becomes asymmetric (middle panel), a feature that we leverage to achieve stable resonator light intensity control (“Methods”). The return laser power (bottom panel) is monitored via the waveguide output (Supplementary Fig. 6). **b** Trap array translocation. For each nSWAT, two translocation heaters control position of the trap array (top panel). To enable long-distance transport, the voltage on each translocation microheater is reset after the array moves by one period of the trapping potential (bottom left panel). The two translocation microheaters operate in a complementary mode such that the total applied power to the two microheaters remains constant, and the resulting measured position of a bead held on nSWAT shows smooth long-distance transport (bottom right plot). **c** Rapid response speed of trap array to the translocation microheaters. To increase the response speed of trap array position to the microheater at the reset, a brief voltage overshoot is applied to translocation microheater 2. To examine the effectiveness of this approach in phase modulation, a high-speed detection method (1 MHz) is required. We thus fabricated a test device, whose schematic is shown in the top panel, to enable this detection (Supplementary Fig. 9). Bottom plots show microheater modulation and the corresponding phase modulation using this device. The characteristic response time constants were obtained from fits to an exponential function (not shown). Source data for this Figure are provided as a Source data file.

To limit the return light, each resonator is designed to operate near its critical-coupling condition (Fig. 2a; Supplementary Fig. 5). When the resonator is tuned to resonance, nearly all light in the bus waveguide is coupled into the resonator, and the return light is greatly minimized. Because the trapping region resides at the resonator, scattering occurs at the boundaries of the etched fluid pool and at the trapped beads, inevitably reducing the quality factor ($Q$) of the resonator. Fortuitously, the resulting resonance broadening reduces the sensitivity of the critical-coupling condition to the separation distance between the bus waveguide and the resonator so that critical coupling can be realized within the tolerance of the fabrication uncertainties. In addition, the resonance condition is also less susceptible to changes in the number of beads trapped on the waveguide.

For each resonator, we measured its $Q$ via the scattered light of the intensity samplers of the resonator and the return light via scattered light at the ‘return’ light sensor (Supplementary Fig. 6; Supplementary Method 2; Supplementary Method 4). As shown in Fig. 2a, as the resonator is tuned towards resonance, the light intensity in the resonator peaks, while the return laser light intensity decreases to a minimum. We found that the phase response curve of a resonator is symmetric at lower input laser powers but becomes asymmetric with an increase in the laser power. This asymmetry is indicative of nonlinearity due to a light-induced thermo-optic effect^36,37^. For our devices, we found that ${Q}$ is ~2 × 10^5^ at 60 mW of input laser power and decreases to ~0.5 × 10^5^ at the 1.3 W of input laser power typically used in subsequent DNA stretching and unzipping experiments (Supplementary Fig. 7).

### Microheater power overshoot to achieve high force

To precisely translocate an array of beads on an nSWAT over a long distance, we ramp the power in a pair of translocation microheaters (Fig. 2b). To translocate over a distance greater than one spatial period of the trapping potential, we rapidly reset the power to each microheater after the trap array has been moved by precisely one period of the trapping potential before re-ramping its power. This process is repeated until the translocation reaches the desired distance. While this method permits long-distance translocation without overheating the microheaters, it may also restrict the force generation capacity if the reset is not sufficiently rapid. During the brief reset, a bead initially trapped may drift in the reverse direction under the influence of the reverse moving trapping potential. This drift becomes more evident if the bead is also experiencing an external force opposing the translocation (e.g., from an attached DNA molecule). Based on analysis of the non-linear dynamics of the bead motion in an array trap^38^ (Supplementary Fig. 8), the slower the reset speed and the higher the external force, the greater the drift distance. If the bead drifts beyond half of the spatial period of the trapping potential, a stable trapping condition can no longer be sustained, and during the reset, the bead will slip along the waveguide.

Thus, rapid reset is crucial for obtaining a high trapping force in a translocating nSWAT. Our analysis shows that if the bead is under an external force of 30 pN, the microheater reset needs to be 2 µs in order to obtain 90% of the maximum trapping force. This reset speed is significantly faster than that previously demonstrated (~30 µs)^32,36^. Although the reset speed can be improved by placing the microheater closer to the waveguide, this solution leads to greater light loss due to increased scattering by the microheater. Instead, we have circumvented this problem by applying a brief overshoot (~2 µs) to one of the translocation microheaters at the beginning of the reset to over-drive this microheater. As shown in Fig. 2c and Supplementary Fig. 9, this method reduces the effective microheater reset time to 1.0 µs (Supplementary Method 7). Therefore, the microheater reset speed is no longer a limiting factor for high force generation.

### Single-molecule applications

**Fig. 3:**
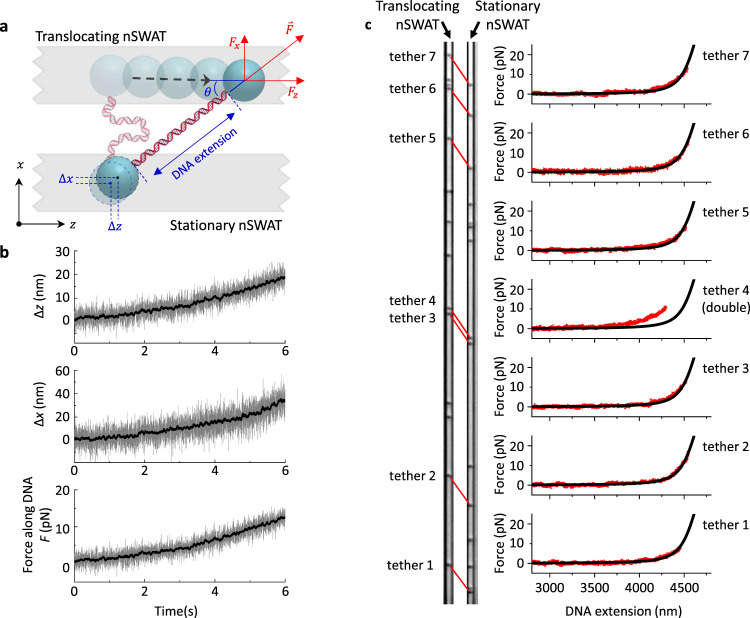
Measuring force-extension curves of DNA on nSWAT. **a** Force diagram of a DNA tether being stretched. A DNA molecule, tethered between two 380-nm polystyrene beads, was stretched by translocating one nSWAT (top) relative to a stationary nSWAT (bottom). The positions of both beads were monitored via image tracking. The total force along the DNA was obtained from force components along $x$ and $z$, using the displacements of the bead in the stationary trap, $\triangle x$ and $\triangle z$ (“Methods”; Supplementary Figs. 11 and 12; Supplementary Method 8). This method of force measurements allows a more accurate determination of force by using both measured $\triangle x$ and $\triangle z$. **b** Example of force measurements of a DNA tether being stretched. Data were taken at 1000 Hz (gray), and smoothed to 20 Hz (black). **c** Simultaneous force-extension measurements of dsDNA molecules. The left panel shows an optical image of seven dsDNA tethers that were simultaneously stretched between a stationary nSWAT and translocating nSWAT. The red lines were manually added to indicate the locations of the dsDNA molecules which are not visible on this image. The resulting measured force-extension curves are shown in the right panel, along with the theoretical prediction (black curves; “Methods”). Each curve is in agreement with that predicted for a single dsDNA molecule, except for tether 4, which is consistent with a double dsDNA tether. Source data for this Figure are provided as a Source data file.

We then evaluated the performance of this device with three standard single-molecule optical trapping experiments. First, we stretched DNA to measure the force-extension relation of DNA^8,9^. Here, we first sorted an array of DNA tethers—single molecules of DNA with a bead attached at each end—from a mixture of other bead species, using a method similar to what has been described^32^ (“Methods”; Supplementary Fig. 10a). Subsequently, we stretched this array of DNA molecules to ~15 pN by translocating one nSWAT relative to another nSWAT held stationary. During the stretch, both $z$ and $x$ positions of all beads in the array were monitored continuously using an image-based tracking technique (“Methods”), providing a direct measurement of the extension (end-to-end distance) of each DNA molecule in the array (Fig. 3a). For each DNA tether, ${F}_{z}$ (or ${F}_{x}$), the force along $\hat{z}$ (or $\hat{x}$), was also determined using the $z$ (or $x$) displacement of the bead held in the stationary nSWAT and the calibrated trap stiffness (Fig. 3b; “Methods”; Supplementary Figs. 11 and 12; Supplementary Method 8). The net force $F$ on the DNA was then determined from these force components. Thus, the force-extension relation, characteristic of DNA elastic properties, was simultaneously measured for each DNA molecule in the array.

Figure 3c shows an experiment where the force-extension relations from seven DNA tethers were obtained in parallel. Six of the seven traces show an agreement with the relation expected for a single dsDNA molecule under the experimental condition used (“Methods”). One trace deviated from expectation and is consistent with a tether formed by two dsDNA molecules instead of one, a configuration that occasionally occurs due to the stochastic nature of the tether formation process. This experiment is akin to DNA stretching using conventional optical tweezers^8,9^, except that manipulation is performed on multiple DNA molecules simultaneously, illustrating the capacity of nSWAT for parallel precision force measurements on-chip.

**Fig. 4:**
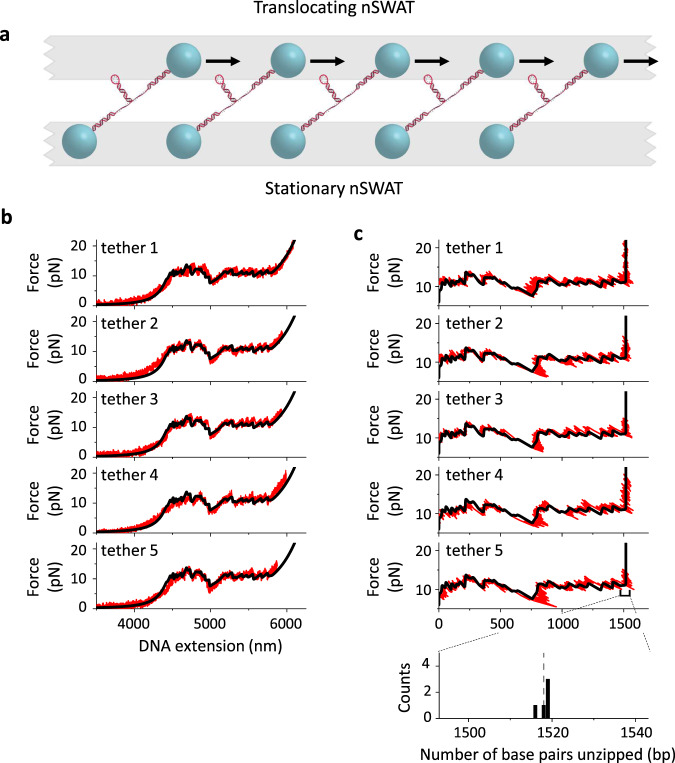
Unzipping DNA molecules on nSWAT. **a** Schematic of the DNA unzipping configuration. Each DNA molecule contained a dsDNA segment to be unzipped with the distal end capped by a DNA hairpin and the two strands attached to two beads via dsDNA adapters. As the bead in the translocating nSWAT was moved away from the bead in the stationary nSWAT, the dsDNA molecule was unzipped. **b** Force versus extension curves (red) of five DNA molecules simultaneously unzipped by nSWAT, together with theoretical prediction (black; “Methods”). **c** Force versus number of base pairs curves converted from traces shown in (**b**) (“Methods”). For each trace, the force rise above the DNA baseline provides a measure of the location of the hairpin. The bottom panel shows a histogram of the detected location of the endpoint of the hairpin from these traces: 1518.0 ± 1.0 bp (mean ± SD), along with the expected location of the hairpin: 1518 bp (vertical dashed line). Source data for this Figure are provided as a Source data file.

Next, we unzipped DNA by mechanically separating a double-stranded DNA (dsDNA) molecule into two single-stranded DNA (ssDNA) molecules^39–41^ (Fig. 4a; Supplementary Fig. 10b). Here, the two strands of dsDNA were suspended between two beads via two dsDNA adapter arms, and the distal end of the dsDNA was capped by a DNA hairpin. As an array of such tethers was simultaneously stretched, the force and extension relation of each DNA molecule in the array was measured concurrently (Fig. 4b). At the beginning of each trace, the force in the DNA was insufficient to unzip the dsDNA, so the force-extension curve followed that of dsDNA from the two arms. Once the arms were extended, and the force rose to ~12 pN, unzipping the dsDNA began with unzipping force fluctuating in a manner fully predictable according to the DNA sequence. When the unzipping fork encountered the end-capped hairpin, the hairpin was fully unfolded, and further stretching extended both the ssDNA and the dsDNA arms, with the endpoint of the hairpin mimicking a strongly bound protein that resists unzipping^20^. For each trace, the measured force-extension curve (Fig. 1b) was correlated with the predicted force and converted to force versus the number of base pairs (bp) unzipped (Fig. 4c), using the elastic properties of dsDNA and ssDNA (Supplementary Method 9). The location of the endpoint of the hairpin was identified by a force rise above the baseline at the end of the trace. We found that the hairpin was located with base-pair accuracy and precision (bottom plot of Fig. 4c), representing the first demonstration of any nanophotonic platforms to achieve a level of performance comparable to that of a conventional precision optical trapping instrument^20^.

**Fig. 5:**
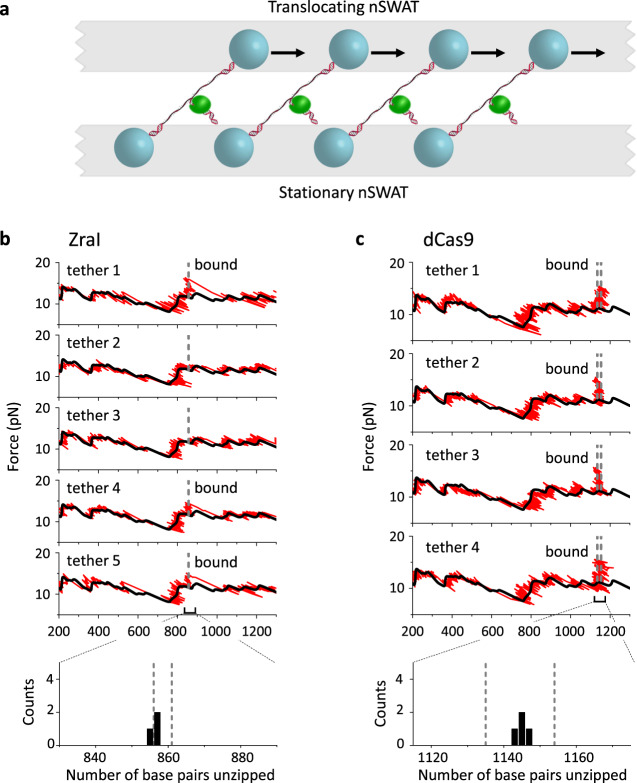
Mapping protein–DNA interactions on nSWAT. **a** Schematic of the DNA unzipping mapper configuration. This experimental configuration is similar to that used in Fig. 4a, except that unzipping was carried out in the presence of a protein that can bind to a specific location on the DNA to be unzipped. **b** Simultaneous mapping of a bound restriction enzyme ZraI on five DNA molecules. For each trace with a bound ZraI, a force rise above the baseline indicates that the unzipping fork encountered a bound ZraI protein and provides a measure of the location of the bound ZraI. The two vertical dashed lines bracket the 6 bp recognition site of ZraI. The bottom panel shows a histogram of the detected location of a bound ZraI from these traces: 856.0 ± 0.6 bp (mean ± SD). **c** Simultaneous mapping of a bound dCas9 on four DNA molecules. The two vertical dashed lines bracket the 20-bp DNA sequence that the sgRNA recognizes when complexed with dCas9. The bottom panel shows a histogram of the detected location of a bound dCas9 from these traces: 1145.0 ± 1.7 bp (mean ± SD). Source data for this Figure are provided as a Source data file.

Finally, we applied the DNA unzipping technique for parallel mapping of protein–DNA interactions on nSWAT (Fig. 5a). Just as with a DNA hairpin, a bound protein on the DNA is also able to resist unzipping, producing a similar force rise when encountered by the unzipping fork^14,18^, a technique also termed ‘unzipping mapper’^21^. We first unzipped an array of dsDNA molecules, each containing a binding site for the restriction enzyme, ZraI, which targets a unique 6-bp DNA sequence. For the dsDNA unzipping segment shown in Fig. 5b, there is a ZraI binding site centered at 859 bp from the start of the unzipping. To enable ZraI binding without its DNA enzymatic cutting activity, the experiment was carried out in the absence of magnesium which is required for cutting. Under the experimental conditions used, we found that a fraction of the traces showed a force signature consistent with a bound ZraI: a force rise was detected at a ZraI binding site, followed by a return of the force to the unzipping baseline after the unzipping fork passed the ZraI site. Thus, the nSWAT generated sufficiently strong forces to disrupt a bound ZraI, with a mean disruption force of 15.7 ± 1.5 pN (mean ± SD).

We then used the unzipping mapper to locate a bound dCas9, which is the non-catalytic mutant of Cas9 and has been broadly used as a strong roadblock for DNA processing enzymes^42^. When dCas9 is complexed with a single guide RNA (sgRNA), it targets a 20-bp DNA sequence complementary to the sgRNA^43^. We designed the sgRNA to target a sequence centered at 1145 bp from the start of the unzipping. We unzipped an array of DNA molecules, each containing a bound dCas9, with the unzipping fork approaching the bound dCas9 from the RNA/DNA hybrid side. For each trace, we detected a force rise within the sgRNA target region, consistent with Cas9 interaction sites with DNA from previous studies^44–46^. Although nSWAT did not disrupt the bound Cas9, nSWAT could still accurately map the Cas9 location because the force was significantly greater than the baseline force. The precision of the detected ZraI binding site was 0.6 bp, similar to that of the hairpin, while the precision of the detected Cas9 binding sites was 1.7 bp, somewhat larger than that of the hairpin, likely due to different conformations that these proteins may adapt upon binding to DNA^47,48^.

## Discussion

Taken together, these applications demonstrate that the resonator-nSWAT platform can perform as a precision optical trapping instrument. Our Si_3_N_4_ resonator-nSWAT platform provides significant trapping force enhancement, generating forces on the order of 20 pN, while allowing for base-pair resolution measurements of multiple molecules at the same time. This has enabled applications of nSWAT in several significant single-molecule optical trapping assays: measuring DNA elasticity, unzipping DNA molecules, and mapping protein–DNA interactions. Thus, this work presents a substantial advancement of the nanophotonic tweezers for future applications of a broad range of biological studies to be performed with nSWAT.

## Methods

### Fabrication protocol for Si_3_N_4_ resonator-nSWAT device

The Si_3_N_4_ resonator-nSWAT utilized deep ultraviolet (DUV) lithography for the waveguide patterning and had an N_2_ annealing process for minimizing the optical loss after the waveguides were plasma etched with SF_6_/CH_2_F_2_/N_2_. Ni microheaters were evaporated on top of the waveguides to modulate the phase of standing-wave array traps. Moreover, several key design changes and improvements to the published Si_3_N_4_ protocol^33^ were made in the development of resonator-nSWAT (Fig. 1; Supplementary Fig. 1).

1. The addition of the resonator design requires additional microheaters to tune the resonance condition of the resonators.
2. To accommodate these additional microheater electronics on the chip, a 3D electronic architecture was implemented with aluminum vias to connect the microheaters to the electronics contact pads.
3. The layout of the waveguides on the resonator-nSWAT was designed to minimize waveguide bending loss. The number of bends was minimized, and the radius of bend curvature was kept at > 80 μm to reduce the bending loss (Fig. 1c).
4. For each resonator-nSWAT device, two local light intensity samplers are coupled to each bus waveguide, and two local light intensity samplers are coupled to each resonator loop. Each intensity sampler is a waveguide that is weakly coupled (1:99) to the main waveguide. At the terminus of each sampler waveguide is a Si_3_N_4_ scattering grating. Using these samplers, the local light intensities traveling in both the clockwise and counterclockwise directions in each nSWAT were detected by an IR camera at a frame rate of 30 Hz. This intensity may also be used in an intensity clamp by a control software to feedback on the applied voltages to the resonator microheaters.
5. To define the perimeter of a flow channel, each chip was patterned with a 15-μm tall and 200-μm wide SU8 barrier wall that encloses the trapping regions of 8 devices. To regulate the flow of UV glue while sealing the flow channel, one 15-μm tall and 100-μm wide SU8 boundary and two 7-μm tall and 100-μm wide SU8 boundaries s are patterned outside the periphery of the flow channel, with 100 μm separations in between (Supplementary Fig. 1).

Detailed information of the device parameters and design calculations are presented in Supplementary Methods 1–4.

### Flow cell assembly

In contrast to the coverslip-parafilm-chip flow cell assembly for previous nSWAT designs^32^, the flow cell assembly protocol for the resonator-nSWAT devices has been significantly improved for better flow cell quality, robustness, and reproducibility. A shallow and well-defined flow cell is critical to bring the biological sample to the resonator-nSWAT devices while also allowing for a high NA, water immersion objective (Nikon MRD07602) to image the trapped beads. To form a flow cell sample chamber on top of the fabricated resonator-nSWAT devices, a #1.5 coverglass is glued with a UV curing optical adhesive (Norland). First, a coverglass is cut to the appropriate size to cover the diced resonator-nSWAT chip. Entry and exit ports are drilled into the coverglass with a sandblaster and Tygon tubes are glued onto the top of the coverglass with UV glue (Norland NOA68). Next, the coverglass is placed onto the resonator-nSWAT chip and held in place by applying a vacuum to the Tygon tubes. The SU8 pattern (Supplementary Fig. 1) provides a seal and defines the flow cell boundary. Small drops of moderate viscosity UV curing glue (Norland NOA86) is applied at several locations around the periphery of the coverglass, and the glue is then pulled inwards by capillary action, which has been shown to facilitate the formation of a flow cell^49^. Before this glue reaches the inner SU8 flow cell boundary, it is cured with UV light. The vacuum is then removed from the flow cell. Lower viscosity glue is then introduced to the periphery of the coverglass and is again pulled in by capillary action. The viscosity of the UV glued is tuned by blending UV glues (Norland NOA86 & NOA87) to allow the glue to be pulled up to the SU8 flow cell boundary without being pulled past the boundary and into the flow cell. When the UV glue has reached the SU8 flow cell boundary, it is cured by UV light.

### Lipid coating procedure

The waveguide surface is passivated to prevent non-specific binding of beads and proteins by coating the flow cell chamber surfaces with a lipid bilayer following a protocol similar to that previously described^32^ with the following changes. A fresh ampoule of 1,2-dioleoyl-sn-glycero-3-phosphocholine (DOPC) in chloroform (Avanti 850375C) is dried by a steady stream of nitrogen gas. The lipids are re-suspended in a high salt buffer (10 mM Tris-HCl pH 8.0, 100 mM NaCl) to a final concentration of 10 mg/mL. The re-suspended lipids are then sonicated for an hour to produce small vesicles. The lipids are diluted to 5 mg/mL by the addition of the high salt buffer. The lipid solution is then introduced to the flow cell and incubated overnight for thorough coverage of lipid around the waveguides. The high salt buffer subsequently flushes out the lipid solution to remove excess lipid inside the flow chamber. After that, a low salt buffer (15 mM Tris-HCl pH 7.8, 0.2 mg/mL BSA, 0.5 mM EDTA, 0.77 mM NaN_3_) is then flowed in to allow lipid annealing for 3 h before subsequent trapping experiments^50^.

### Preparation of DNA

#### Stretching template

The 13,709 bp dsDNA stretching template is made via PCR from λ DNA using LongAmp DNA polymerase, a 5′ biotin-labeled forward primer 5′-GCT GAT GCT TGA ACC CGC CTA TGC, and a 5′ digoxigenin-labeled reverse primer 5′-CTC AGT TTG CCT TCC TCC GTG TCC TC. The resulting DNA was purified with an Invitrogen PureLink PCR purification kit (Invitrogen K310001).

#### Unzipping template

The unzipping template was made based on a previous method^40^. Arm 1 DNA and arm 2 DNA have identical sequences (6.8 kbp), which were cut from plasmid pRL574. The 5′ ends of arm 1 and arm 2 were labeled with digoxigenin and biotin through PCR reaction, respectively. Each arm was cut with DraIII (NEB), leaving a 3′ overhang (CAT), and was then ligated to an adapter DNA (upper1: 5′-/5Phos/GCA GTA CCG AGC TCA TCC AAT TCT ACA TGC CGC, lower1: 5′-/5Phos/GCC TTG CAC GTG ATT ACG AGA TAT CGA TGA TTG CGG CGG CAT GTA GAA TTG GAT GAG CTC GGT ACT GCT AC for arm 1; upper2: 5′-CGT TAC GTC ATT CTA TAC ACT GTA CAG TAC, lower2: 5′-/5Phos/CTG TAC AGT GTA TAG AAT GAC GTA ACG CGC AAT CAT CGA TAT CTC GTA ATC ACG TGC AAG GCC TA for arm 2). Each ligated arm is gel purified to remove un-ligated adapters. Then, arm 1 and arm 2 were annealed via the two adapter DNAs, forming the start of the unzipping DNA segment. The rest of the unzipping segment (1.5 kbp) was cut from plasmid pRL574, at one end with AlwNI (NEB) for ligation to the start of the unzipping segment and at the other end with BstEII (NEB) for ligation to a 6T hairpin. The total length of the unzipping segment is 1515 bp without counting the hairpin (Supplementary Fig. 10). The final ligation product was gel purified using Zymo Large Fragment DNA Recovery Kit (D4045).

### Data acquisition and bead tracking

The nSWAT data collection software is implemented in LabVIEW 2017. Trapped beads are imaged by a high NA water immersion objective onto a high-speed CMOS camera (JAI-GO-2400M-USB). The camera region of interest is set to allow a frame rate of 1000 fps, which is used for power spectrum data collection, DNA stretching, and unzipping experiments. This image data is processed in the nSWAT data analysis software implemented in LabVIEW 2020. For each image frame, the waveguide background is subtracted, and a complement image is used for bead tracking.

Each bead in a camera frame is tracked by performing a 2D Gaussian fit to the bead image^51^. To compensate for sample drift, fiducial markers that have been fabricated to locate adjacent to the waveguides in the fluid pool region and are also tracked during the stiffness calibration step (Supplementary Fig. 11). Although the direct 2D Gaussian fitting method gives high tracking accuracy, it requires the object to have a Gaussian intensity profile and thus cannot be used to track the fiducial marks. Thus, the fiducial markers are tracked by a cross-correlation-based method with the kernel image being a direct fitting of the fiducial marker^52^.

On the optical setup, a dichroic mirror is placed in the imaging path to collect IR light onto an IR camera (SU320HX-1.7RT). The IR camera acquires images of the fluid pool region during each experiment at 30 fps to record scattered trapping light. The four IR light scatters of each nSWAT measure the real-time trapping laser intensity at the resonator (Fig. 2a; Supplementary Fig. 11) and input laser intensity at the bus waveguides.

Detailed information of trap stiffness calibration and force determination based on the tracked bead trajectory along with the recorded IR light intensity is presented in Supplementary Method 8.

### Preparation of beads and DNA-tethers

To prepare beads used for the experiments, we started with 380 nm carboxylated polystyrene beads from Polysciences (CAT#21753), which had a guaranteed 3% coefficient of variation in the bead diameter. We then coated these beads with either anti-digoxigenin (Roche #11333089001) or streptavidin (Agilent SA10) following protocols from the Polysciences. The anti-digoxigenin was dissolved in 0.2 M Borate acid buffer pH 8.5 at the concentration of 2 mg/mL and then used in the bead coating process.

For the formation of DNA tethers, i.e., single molecules of DNA with a bead attached at each end, DNA-bead binding took place in the binding buffer (50 mM Tris-HCl pH 7.8, 100 mM NaCl, 0.1 mg/mL BSA). A DNA template was first mixed with anti-digoxigenin-coated beads and incubated for 20 min, followed by the addition of streptavidin-coated beads with an additional incubation of 10 min. The ratios of DNA template, anti-digoxigenin-coated beads, and streptavidin-coated beads were 1:0.9:0.8 with the DNA template concentration at 100 pM. The DNA-bead mixture was then diluted into the low salt buffer (15 mM Tris-HCl pH 7.8, 0.2 mg/mL BSA, 0.5 mM EDTA, 0.77 mM NaN_3_) at 1:10 dilution then stored on a rotor at 4 °C, serving as DNA tether stock. Immediately before introducing into an nSWAT chamber for measurement, the DNA-bead mixture was further diluted at 1:10 in the low salt buffer, leading to a final DNA concentration during an nSWAT measurement of 1 pM.

### Formation of protein–DNA complexes

#### Restriction enzyme ZraI

Restriction enzyme ZraI (NEB R0659L) was first concentrated from 10,000 to 100,000 unit/μL using Amicon Centrifugal Filters (UFC501008) at 4 °C and then stored at −20 °C for future use. Before an nSWAT measurement, this concentrated ZraI was added to the unzipping DNA stock at 1:200 dilution, and this mixture was incubated at room temperature for 30 min.

#### dCas9

The sgRNA target sequence (GCGCGUAUCAUCCCUUACCG) was cloned into a pUC19 vector containing the sgRNA scaffold followed by an HDV ribozyme. A PCR product containing the sgRNA T7 transcription template was created from this plasmid and was used for in vitro transcription of the sgRNA using T7 RNA polymerase (NEB M0251S). The sgRNA was then purified using denaturing PAGE and stored in TE pH 8.0 (Invitrogen) at −80 °C until use. To bind dCas9 to the unzipping template, we mixed the sgRNA and dCas9 (NEB M0652S) at 1 nM in the binding buffer (50 mM Tris-HCl pH 7.8, 1 mM MgCl_2_, 0.1 mg/ml BSA, 100 mM NaCl) and then added to the unzipping template at 100 pM. This mixture was incubated at 37 °C for 30 min. After incubation, the DNA template with dCas9 bound was then used to form DNA-tethers following protocols described above.

### Trapping and sorting procedure

Trapping and sorting of DNA tethers on an nSWAT were based on the previous method of alternating the fluid flow direction and concurrently modulating the trap power on and off^1^ with the following modifications. The resonator microheaters were used to control the on and off states of each nSWAT. Since the resonance peak of the resonance curve changes with the number of beads trapped on an nSWAT, we implemented a self-adaptive microheater current optimization method to maximize the trapping light intensity in the resonator. Both trapping and sorting were operated at a low laser power input to the device (~150 mW).

In this method, the tether orientation was not controlled so that a streptavidin-coated bead in a DNA tether could be trapped on either the top or bottom nSWAT. This orientation degeneracy should not affect the measured force and extension of a DNA molecule.

### Stretching and unzipping of DNA

DNA stretching and unzipping experiments were operated at a high laser power input to the device (~1.5 W). Such a high laser power induces a strong thermo-optic effect within the resonator and shifts its resonance peak of the resonance curve (Fig. 2a). Thus, to increase the laser power to this high laser power, we implemented a dynamic resonator microheater tuning method to maintain a near-resonance condition.

To minimize the impact of the imbalance in the 50/50 splitters on trapping force generation, the waveguide with a higher trapping stiffness was selected as the stationary nSWAT, and this ensured reliable tracking of beads on this nSWAT. To stretch/unzip DNA, the translocating nSWAT was moved under a constant speed in the direction of a stronger scattering force. During this process, the image of the region of interest was recorded at 1000 fps by the CMOS camera, while the IR camera simultaneously recorded the light intensities at the scatterers at 30 fps. The force-extension relation of each tether was obtained by bead tracking of these video images (see description above as well as Fig. 3 and Supplementary Fig. 11).

To convert the force-extension relation of an unzipping trace to the number of base pairs of DNA unzipped, we performed the following data processing. First, the force-dependent contribution to the extension by the 13,576 base pairs of dsDNA arms was subtracted from the overall DNA extension to obtain an extension that was solely due to the ssDNA. Next, a small shift and stretch term was applied to the ssDNA extension to align the DNA baseline force signature to the expected theoretical prediction^19,39^. The shift and stretch parameters were determined for each data trace by maximizing the weighted normalized cross-correlation between the measured and expected force versus ssDNA extension curves. The corrected ssDNA extension and measured force were then used to calculate the number of ssDNA nucleotides from the freely-jointed chain model^8^. The number of base pairs unzipped was one-half of the number of ssDNA nucleotides. The dsDNA and ssDNA parameters used in this calculation were measured using aforementioned methods.

For Fig. 5, a bound protein was detected at location with a force rise of at least 2.5 pN above the naked DNA unzipping baseline.

### Reporting summary

Further information on research design is available in the Nature Research Reporting Summary linked to this article.

## Supplementary information

Supplementary Information

Description of Additional Supplementary Files

Supplementary Movie 1

Supplementary Movie 2

Supplementary Movie 3

Reporting Summary

## Data Availability

Relevant source data for the main text and Supplementary Figures are provided in the Source Data section. All other data that support the findings of this study are available from the corresponding author upon reasonable request. Source data are provided with this paper.

## Source data

Source Data

## Code availability

All relevant code is available upon reasonable request.
