# Supplementary material for: Resonator nanophotonic standing-wave array trap for single-molecule manipulation and measurement
Source: Nat Commun. 2022 Jan 10;13:77. doi: 10.1038/s41467-021-27709-3 (PMC8748738; doi:10.1038/s41467-021-27709-3)
Supplement: Supplementary file 1 — Supplementary Information [file 41467_2021_27709_MOESM1_ESM.pdf]

Supplementary Information:

Resonator Nanophotonic Standing-Wave Array Trap for Single-Molecule Manipulation and Measurement

Fan Ye, James T. Inman, Yifeng Hong, Porter M. Hall, and Michelle D. Wang

Table of Content:

- **Supplementary Methods 1-9**
- **Supplementary Figures 1-12**

Supplementary Methods

1. Device Parameters

In this section, we provide device parameters related to the nanophotonic aspect of the resonator-nSWAT. The trapping laser wavelength is λ_0 = 1.064 µm. The Si_3N_4 waveguide has a cross-section of 750 nm x 250 nm, embedded in SiO_2. The fundamental TM-like mode is guided in the Si_3N_4 waveguide to achieve higher manipulation forces to trapped beads[1]. The effective index of refraction (n_{eff}) and the group index of refraction (n_{g}) of this TM-like mode at 1.064 µm is 1.57 and 1.91, respectively (Supplementary Fig. 2). Group index is calculated using: $n_{\mathrm{g}} = n_{\mathrm{eff}} - \lambda_0 \frac{dn_{\mathrm{eff}}}{d\lambda}$. The total waveguide length of the resonator loop is L = 8600 µm, including a 110 µm long fluid pool section where the top surface of the Si_3N_4 waveguide is exposed to water. The free spectral range (FSR) of the resonator is thus: $\mathrm{FSR}_f = \frac{c}{n_{\mathrm{g}}L}$ = 18.3 GHz or $\mathrm{FSR}_\lambda = \frac{\lambda_0^2}{n_{\mathrm{g}}L}$ = 69 pm. The dispersion of Si_3N_4 and SiO_2 materials are taken into account using the Sellmier equation: $n^2 - 1 = \frac{A_1\lambda^2}{\lambda^2 - B_1^2} + \frac{A_2\lambda^2}{\lambda^2 - B_2^2}$, with the coefficients for Si_3N_4 [1] set as: A_1 = 3.0249, A_2 = 40314, B_1 = 0.1353406, B_2 = 1239.842, and the coefficients for SiO_2 [2] set as: A_1 = 1.1445, B_1 = 0.0874, A_2 = B_2 = 0.

In the fluid pool region, two copies of the nSWAT device are brought close to each other to perform parallel single-molecule manipulations. The center-to-center separation between the two parallel waveguides in the fluid pool region is 2.6 µm, corresponding to an effective index of refraction difference Δn_{eff} = 0.000071 between the symmetric and anti-symmetric modes. The index of refraction of water at 1.064 µm is set as 1.3239[3]. For a 110 µm long fluid pool region (L_{fp}), the energy crosstalk between two copies of the nSWAT can be calculated to be: $\frac{1}{2}\left[1 - cos\left(\Delta n_{\mathrm{eff}} \frac{2\pi}{\lambda_0} L_{\mathrm{fp}}\right)\right]$= 0.05% (Supplementary Fig. 3). This corresponds to minimal expected crosstalk between the two waveguides. We also did not observe any detectable crosstalk between the two copies of the nSWAT in our experiments.

2. Return laser power

In an nSWAT device, some of the light input to the device may be ultimately returned to the waveguide input by retracing the same path as that of the input light. This section describes a method for monitoring this return laser power.

Consider a single copy of resonator-nSWAT (Supplementary Fig. 6). For a perfect 50/50 splitter, light will be eventually returned to the input port (port 1), without any light exiting port 2. In reality, the 50/50 splitter is often slightly imbalanced, and in that case, some light also exits port 2. Below, we show that the output light at port 2 is proportional to this return laser power and thus can be used for monitoring the return laser power.

Here, we denote the electrical fields of laser inputs (and outputs) of the 4 ports of the splitter as I_i (and O_i), $i = 1, 2, 3, 4$, with port 1 being the incident waveguide of the nSWAT device. We also denote the loss coefficient and phase change of half the bus-waveguide loop where translocation microheaters are located as α_t and φ_t respectively. Furthermore, we denote the input and output electrical fields of the resonator as a and b, and thus,

$$\frac{b}{a} = \frac{t - \alpha_\mathrm{r} e^{i\varphi_\mathrm{r}}}{1 - \alpha_\mathrm{r} t e^{i\varphi_\mathrm{r}}},$$

where t is the transmission coefficient of the resonator coupler, α_r is the loss coefficient of the resonator loop, φ_r is the phase change of the resonator loop.

We then have the relation between I_3 and O_4, and between I_4 and O_3:

$$I_3 = \frac{t - \alpha_\mathrm{r} e^{i\varphi_\mathrm{r}}}{1 - \alpha_\mathrm{r} t e^{i\varphi_\mathrm{r}}} \alpha_\mathrm{t}^2 e^{2i\varphi_\mathrm{t}} O_4 \equiv \alpha_\mathrm{T} e^{i\varphi_\mathrm{T}} O_4 ,$$

$$I_4 = \frac{t - \alpha_\mathrm{r} e^{i\varphi_\mathrm{r}}}{1 - \alpha_\mathrm{r} t e^{i\varphi_\mathrm{r}}} \alpha_\mathrm{t}^2 e^{2i\varphi_\mathrm{t}} O_3 \equiv \alpha_\mathrm{T} e^{i\varphi_\mathrm{T}} O_3 ,$$

where $\alpha_\mathrm{T} \equiv \frac{t - \alpha_\mathrm{r} e^{i\varphi_\mathrm{r}}}{1 - \alpha_\mathrm{r} t e^{i\varphi_\mathrm{r}}} \alpha_\mathrm{t}^2$, and $\varphi_\mathrm{T} \equiv 2\varphi_\mathrm{t}$.

Under unit incident electric field from I_1, we have the following relation:

$$\begin{bmatrix} I_1 \\ I_2 \\ I_3 \\ I_4 \end{bmatrix} = \begin{bmatrix} 1 \\ 0 \\ \alpha_\mathrm{T} e^{i\varphi_\mathrm{T}} O_4 \\ \alpha_\mathrm{T} e^{i\varphi_\mathrm{T}} O_3 \end{bmatrix}.$$

In general, for parallel waveguide couplers:

$$\begin{bmatrix} O_1 \\ O_2 \\ O_3 \\ O_4 \end{bmatrix} = e^{i\beta L} \begin{bmatrix} 0 & 0 & \cos(\theta/2) & i\sin(\theta/2) \\ 0 & 0 & i\sin(\theta/2) & \cos(\theta/2) \\ \cos(\theta/2) & i\sin(\theta/2) & 0 & 0 \\ i\sin(\theta/2) & \cos(\theta/2) & 0 & 0 \end{bmatrix} \begin{bmatrix} I_1 \\ I_2 \\ I_3 \\ I_4 \end{bmatrix},$$

with $\theta \equiv \Delta\beta L$, where β is the propagation constant in a single waveguide, $\Delta\beta$ is the difference between the symmetric and anti-symmetric modes in the two coupled waveguides, and L is the length of the total coupling region.

Plug in $I_1 = 1$, $I_2 = 0$, $I_3 = \alpha_\mathrm{T} e^{i\varphi_\mathrm{T}} O_4$ and $I_4 = \alpha_\mathrm{T} e^{i\varphi_\mathrm{T}} O_3$, we get:

$$\begin{bmatrix} O_1 \\ O_2 \\ O_3 \\ O_4 \end{bmatrix} = e^{i\beta L} \begin{bmatrix} i \sin\theta \, \alpha_{\mathrm{T}} e^{i\varphi_{\mathrm{T}}} \\ \cos\theta \, \alpha_{\mathrm{T}} e^{i\varphi_{\mathrm{T}}} \\ \cos(\theta/2) \\ i \sin(\theta/2) \end{bmatrix}.$$

So, the output power can be expressed as:

$$\begin{bmatrix} |O_1|^2 \\ |O_2|^2 \\ |O_3|^2 \\ |O_4|^2 \end{bmatrix} = \begin{bmatrix} |\alpha_{\mathrm{T}}|^2 \sin^2\theta \\ |\alpha_{\mathrm{T}}|^2 \cos^2\theta \\ \cos^2(\theta/2) \\ \sin^2(\theta/2) \end{bmatrix}.$$

For perfect 50/50 splitting, $\theta = \pi/2$,

$$\begin{bmatrix} |O_1|^2 \\ |O_2|^2 \\ |O_3|^2 \\ |O_4|^2 \end{bmatrix} = \begin{bmatrix} |\alpha_{\mathrm{T}}|^2 \\ 0 \\ 1/2 \\ 1/2 \end{bmatrix}.$$

Thus, there is no light exiting port 2.

In contrast, for imperfect splitting with a small phase detune Δ, $\theta = \frac{\pi}{2} + \Delta$,

$$\begin{bmatrix} |O_1|^2 \\ |O_2|^2 \\ |O_3|^2 \\ |O_4|^2 \end{bmatrix} = \begin{bmatrix} |\alpha_{\mathrm{T}}|^2 \cos^2\Delta \\ |\alpha_{\mathrm{T}}|^2 \sin^2\Delta \\ (1+\sin\Delta)/2 \\ (1-\sin\Delta)/2 \end{bmatrix}.$$

Thus, output power to the two waveguide ports are proportional to each other:

$$\frac{|O_2|^2}{|O_1|^2} = \tan^2(\Delta)\,.$$

We can thus monitor the returned laser power to the incident waveguide $|O_1|^2$ by measuring the output power of $|O_2|^2$.

3. Coupling coefficients of the resonator

To minimize the undesirable return laser power and to maximize the resonator enhancement of the laser power, it is crucial for the resonator to work at a condition close to the critical-coupling condition. To achieve this goal, we fabricated resonator-nSWAT devices with a variable coupling gap for the resonator coupler with all other dimensions unchanged. We then selected the gap size that gave the highest resonator enhancement and lowest return laser power. In

this section, we calculate the coupling coefficients of the optimized resonator nSWAT from measured resonance response curves.

Denote E_i ($i = 1,2,3,4$) as the complex electric field amplitudes of the 4 ports of the coupling region of the resonator loop (Supplementary Fig. 5). We have the following relations:

$$E_2 = tE_1 + ikE_3,$$

$$E_4 = tE_3 + ikE_1,$$

$$E_3 = \alpha_r e^{i\varphi_r} E_4,$$

where $t^2 + k^2 = 1$,

t: self-coupling coefficient.

k: cross-coupling coefficient.

α_r: single-pass amplitude transmission coefficient of the resonator loop.

$\varphi_r = \frac{2\pi L}{\lambda_{\text{eff}}} = \frac{2\pi n_{\text{eff}} L}{\lambda_0}$: single-pass phase shift of the resonator loop.

L = 8600 µm: round trip length of resonator loop.

$\lambda_{\text{eff}} = \frac{\lambda_0}{n_{\text{eff}}}$ = 0.68 µm: effective wavelength of the TM-like mode in waveguide.

n_{eff} = 1.57: effective refractive index of the TM-like mode in waveguide.

λ_0 = 1.064 µm: wavelength at vacuum of incident laser.

We can derive the relation between the input and output laser intensities of the bus waveguide as:

$$|E_2|^2 = \frac{\alpha_r^2 + t^2 - 2\alpha_r t \cos\varphi_r}{1 + \alpha_r^2 t^2 - 2\alpha_r t \cos\varphi_r} |E_1|^2.$$

We can thus calculate the extinction ratio (ER) as:

$$\text{ER} \equiv \frac{\text{Max}(|E_2|^2)}{\text{Min}(|E_2|^2)} = \frac{(\alpha_r + t)^2}{(\alpha_r - t)^2} \frac{(1 - \alpha_r t)^2}{(1 + \alpha_r t)^2}.$$

Since the return laser power P_{R} is proportional to $|E_2|^2$, ER can thus be obtained by the return power measurement:

$$\text{ER} \equiv \frac{\text{Max}(|E_2|^2)}{\text{Min}(|E_2|^2)} = \frac{\text{Max}(P_{\text{R}})}{\text{Min}(P_{\text{R}})}.$$

According to Bogaerts et al.[4], Finesse can be expressed as:

$$\text{Finesse} = \frac{\text{FSR}}{\text{FWHM}} = \frac{\pi\sqrt{\alpha_r t}}{1-\alpha_r t}.$$

Thus, from the measured ER and Finesse, we can calculate values of α_r and t. Critical coupling occurs when $\alpha_r = t$. Under this condition, $\text{ER} \to \infty$ and Finesse reaches a maximum.

Supplementary Fig. 7a shows an example of a measured resonance response curve of the laser power at the flood pool at 0.06 W input laser power, from which Finesse is measured to be: Finesse = 12.89. The bottom panel of Supplementary Fig. 5b shows the resonance curve of the return power for the same device at 0.06 W input laser power, from which ER is measured to be: ER = 30.92. We thus get: (α_r, t) = (0.91, 0.87), corresponding to slight under coupling condition; or (α_r, t) = (0.87, 0.91), corresponding to a slightly over coupling condition. Both scenarios are very close to the critical-coupling condition.

Another method to estimate α_r and t is to fit the resonance curves of the local laser intensity and the return power. The laser intensity at the fluid pool region is:

$$I_{\text{FP}} = |\sqrt{\alpha_r}E_4|^2 = \frac{\alpha_r(1-t^2)}{1+\alpha_r^2 t^2 - 2\alpha_r t\cos\varphi_r}|E_1|^2.$$

Fitting this laser intensity by: $I_{\text{FP}} = \frac{I_0}{1+\alpha_r^2 t^2 - 2\alpha_r t\cos(\varphi_r - \varphi_0)}$ with I_0 being a proportionality constant and φ_0 the phase at the peak of resonance (top panel of Supplementary Fig. 5b), we obtain $\alpha_r t$ = 0.81. This agrees very well with the above calculation using ER and Finesse, which gives $\alpha_r t$ = 0.80.

We can also obtain values for α_r and t by fitting the return power resonance curve by: $P_{\text{R}} = P_0\frac{\alpha_r^2 + t^2 - 2\alpha_r t\cos(\varphi_r - \varphi_0)}{1+\alpha_r^2 t^2 - 2\alpha_r t\cos(\varphi_r - \varphi_0)}$, with P_0 being a proportionality constant (bottom panel of Supplementary Fig. 5b). This fitting yields: (α_r, t) = (0.91, 0.86) or (0.86, 0.91), which is again consistent with the above results.

All of the above results consistently show that the resonator-nSWAT device under investigation is working at a condition very close to critical coupling.

4. Determination of the Q factor of the resonator

The quality (Q) factor of the resonator can be calculated using the measured local light intensity at the fluid pool region as we tune the resonator microheaters (Fig. 2a, middle section). At a low input laser power (0.06 W), the resonance curve is close to being symmetric, as shown in Supplementary Fig. 7 (see also Fig. 2a). From this measurement, we can calculate the finesse of the resonator $\text{Finesse} = \frac{\text{FSR}}{\text{FWHM}}$, and then the Q factor using[4]: $Q = \frac{n_{\text{g}}L}{\lambda_0}\cdot\text{Finesse}$. For 0.06 W input laser power, the measured finesse is 12.89, corresponding to a Q factor of 2.0 x 10^5. At

high input powers, the light induced thermo-optic effect leads to broadening of the resonance peak (enlarging FWHM) while keeping the FSR unchanged, thus reducing the finesse and the Q factor. This effect is discussed in more details in the reference[4]. Consequently, the measured finesse and Q factor indeed decrease as input laser power increases (Supplementary Fig. 7).

5. Thermal drift reduction

To facilitate high-precision image tracking of the trapped beads, the image of the trapping region on an nSWAT chip must remain focused. However, during an experiment, heat is introduced to the chip during modulation of the microheaters, resulting in thermal expansion of the chip and therefore drift of the focused region. To minimize this drift, we have used the following method for heat dissipation. We have machined the sample stage out of a large aluminum block (27 mm x 58 mm x 80 mm) and suspended it from the instrument by two glass connections (Supplementary Fig. 4a). The glass connections provide thermal insulation from the rest of the instrument, while the large aluminum block serves as a heat sink. An nSWAT chip is then placed onto the sample stage by a custom-made vacuum chuck.

To estimate the temperature rise due to the microheaters, we performed a COMSOL simulation (Supplementary Fig. 4a). The simulation shows that the temperature rise at the fluid pool region is ~ 1.3 ^{o}C above the ambient temperature. The temperature across this flood pool region is highly uniform, with only 0.014 ^{o}C temperature difference across this region.

We then measured the vertical stage drift with and without the suspended stage (Supplementary Fig. 4c). Our data show that the suspended sample stage efficiently reduces the focal drift of an nSWAT chip to less than 200 nm during microheater modulation.

In addition, during a typical stretching or unzipping measurement, the total power applied power to the two microheaters of each nSWAT remains constant, and the power applied to the microheaters of each nSWAT is also tuned to near the resonance and then kept constant. These settings further ensure the thermal stability of nSWAT devices during measurement.

6. Gravity flow cell

A gravity feed system is used to control the flow in the nSWAT flow cell. The gravity feed system consists of two reservoirs (each made by a 2 mL plastic vial) at an elevated height with two Tygon tubings connecting to the entry and exit ports of a resonator-nSWAT chip on the sample stage. One of the two reservoirs is mounted on a vertical translational stage controlled by a stepper motor. The stepper motor is controlled by a home-built control system to tune the relative height between the two reservoirs. This gravity flow cell control system enables accurate and responsive control of flow speed in the nSWAT trapping region, especially in the low-speed regime: -100 μm/s to 100 μm/s.

7. Test device for the reset speed of translocation microheaters

To detect the speed of the trap array relocation by translocation microheaters, a test device was fabricated (Fig. 2c). The test device consists of a Mach-Zehnder interferometer (MZI) controlled by two microheaters (Supplementary Fig. 9b) that were fabricated to have the same dimensions to those of the nSWAT translocation microheaters (Supplementary Fig. 9a). As the analysis below shows, the output from this test device can be used to assess the speed of the trap array relocation by translocation microheaters in a resonator-nSWAT device.

In this section, we use I to denote the electrical field at an input and O to denote the electrical field at an output. For a resonator-nSWAT device (Supplementary Fig. 9a), under unit incident electric field (I_1=1 and I_2=0), and a phase tuning difference $\Delta\varphi_{\mathrm{t}}$ between the two translocation microheaters, we have the following relation:

$$\begin{bmatrix} I_1 \\ I_2 \\ I_5 \\ I_6 \end{bmatrix} = \begin{bmatrix} 1 \\ 0 \\ \alpha_{\mathrm{t}} e^{i\varphi_{\mathrm{t}}} O_3 \\ \alpha_{\mathrm{t}} e^{i\varphi_{\mathrm{t}}+i\Delta\varphi_t} O_4 \end{bmatrix},$$

where α_{t} is the loss coefficient in one arm of the MZI, and φ_{t} is the phase change of that arm. Using the relation between (I_1, I_2) and (O_3, O_4) for a 50/50 splitter:

$$\begin{bmatrix} O_3 \\ O_4 \end{bmatrix} = \frac{\sqrt{2}}{2} e^{i\beta L} \begin{bmatrix} 1 & i \\ i & 1 \end{bmatrix} \begin{bmatrix} I_1 \\ I_2 \end{bmatrix},$$

and the relation between (I_5, I_6) and (O_7, O_8) for the resonator coupler:

$$\begin{bmatrix} O_7 \\ O_8 \end{bmatrix} = i \sin\left(\frac{\theta_{\mathrm{r}}}{2}\right) e^{i\beta L_{\mathrm{r}}} \begin{bmatrix} I_6 \\ I_5 \end{bmatrix},$$

where L_{r} is the coupling length of the resonator coupler, and $\theta_{\mathrm{r}} \equiv \Delta\beta L_{\mathrm{r}}$, we have:

$$\begin{bmatrix} O_7 \\ O_8 \end{bmatrix} = Re^{i\theta} \begin{bmatrix} ie^{i\Delta\varphi_t} \\ 1 \end{bmatrix},$$

where $Re^{i\theta} \equiv i\alpha_{\mathrm{t}} \frac{\sqrt{2}}{2} \sin\left(\frac{\theta_{\mathrm{r}}}{2}\right) e^{i\beta L + i\beta L_{\mathrm{r}} + i\varphi_{\mathrm{t}}}$.

Thus the time-averaged light intensity of the standing wave at position P_0 in the fluid pool is proportional to:

$$\left|\sqrt{\alpha_{\mathrm{r}}} e^{i\varphi_{\mathrm{r}}/2} e^{i\beta z} O_7 + \sqrt{\alpha_{\mathrm{r}}} e^{i\varphi_{\mathrm{r}}/2} e^{-i\beta z} O_8\right|^2 = A\left(1 + \cos\left(2\beta z + \frac{\pi}{2} + \Delta\varphi_t\right)\right),$$

where $A \equiv |\alpha_{\mathrm{r}} R|^2$. For position P_0 located at an antinode before tuning, the light intensity is proportional to: $A(1 + \cos\Delta\varphi_t)$.

For the test device (Supplementary Fig. 9b), the laser power output from port 1 or "output 1", is proportional to $|O_8|^2$ and is directly coupled to a tapered fiber connected to a balanced photodetector (Thorlabs PDB440C). When the same input power and phase tunings from

translocation microheaters are present at the MZI, (I_1, I_2), (O_3, O_4), and (I_5, I_6) remain the same, while (O_7, O_8) depends on the length of the 50/50 coupling region L:

$$\begin{bmatrix} O_7 \\ O_8 \end{bmatrix} = \frac{\sqrt{2}}{2} e^{i\beta L} \begin{bmatrix} 1 & i \\ i & 1 \end{bmatrix} \begin{bmatrix} I_5 \\ I_6 \end{bmatrix}.$$

Plugging in I_5 and I_6, we get:

$$\begin{bmatrix} O_7 \\ O_8 \end{bmatrix} = \frac{1}{2} \alpha_{\mathrm{t}} e^{i\varphi_{\mathrm{t}}+2i\beta L} \begin{bmatrix} 1 - e^{i\Delta\varphi_t} \\ i + ie^{i\Delta\varphi_t} \end{bmatrix}.$$

The output laser power at port 1 is proportional to:

$$|O_8|^2 = \frac{1}{2} |\alpha_{\mathrm{t}}|^2 (1 + \cos\Delta\varphi_t).$$

Therefore, the dependence on phase tuning difference $\Delta\varphi_{\mathrm{t}}$ between translocation microheaters for the standing wave position of a resonator nSWAT and for the output of a MZI shares the same form (Supplementary Figs. 9c and 9d). We can thus use the test device to measure the response time of translocation microheaters in a resonator-nSWAT.

8. Trap stiffness calibration and force determination

The trap stiffness along the waveguide (z axis) is calibrated by recording the Brownian motion of beads trapped on a resonator-nSWAT as a function of the trapping laser intensity. The two scatters of the light intensity samplers of the resonator measures the laser intensity traveling in the clockwise (grating 1) and counterclockwise (grating 2) directions (Supplementary Fig. 11a and 11b). Thus, the standing wave intensity is estimated to be proportional to the average of these two scattering signals. The trajectories of the trapped beads are analyzed using the power spectrum method, including a correction for blurring and aliasing introduced by the camera[5]. Briefly, the resampled power spectrum is fitted by a modified Lorentzian function

$$P(f) = \sum_{n=-\infty}^{+\infty} \frac{\frac{k_{\mathrm{B}}T}{2\pi^2\beta}}{f_{\mathrm{c}}^2 + (f + nf_{\mathrm{s}})^2} \times \left(\frac{\sin(\pi(f + nf_{\mathrm{s}})W)}{\pi(f + nf_{\mathrm{s}})W} \right)^2 + \frac{\varepsilon^2}{f_{\mathrm{s}}},$$

where $k_{\mathrm{B}}T$ is the thermal energy, f_{c} is the corner frequency, f_{s} is the sampling frequency, W is the camera integration time, β is the drag coefficient, and ε^2 is the inherent position detection error. Fitting this modified Lorentzian function (truncating at $n = 4$) yields f_{c}, β, and ε^2 (Supplementary Fig. 11d). The trap stiffness, k is then calculated by: $k = 2\pi\beta f_{\mathrm{c}}$.

For each nSWAT device, the average trap stiffness for a mixture of the anti-dig and streptavidin-coated 380 nm polystyrene beads is measured at several laser powers. The trap stiffness is plotted versus the average IR intensity from the scattering gratings, and a linear fit gives the stiffness per unit intensity (Supplementary Fig. 11e).

The force exerted on the bead from the resonator-nSWAT trap during an experiment is determined by using a force map generated from finite element COMSOL simulations and the trap stiffness measured above. A 3D COMSOL simulation was performed for a 380 nm polystyrene sphere at 5 nm above a 250 nm × 750 nm Si_3N_4 waveguide as a function of x and z displacements from the trap center at 1 W trapping power (defined as the sum of powers in two directions) (Supplementary Fig. 12). This map is then scaled based on the measured z stiffness per unit intensity. Subsequently, the measured Δx and Δz displacements of the bead and the local intensity are then used to look up the F_x and F_x forces exerted on the bead by the trap from the force map. The resulting force along the tether can be calculated by $F = F_x \sin\theta + F_z \cos\theta$, where θ is the measured angle of the DNA tether relative the nSWAT waveguides (Supplementary Fig. 12; Fig. 3). Force along the tether can also be calculated from a single force component alone, by $F = F_x/\sin\theta$ or $F = F_z/\cos\theta$, but with amplified noises at θ values close to 0 or $\pi/2$, respectively. The vast majority of traces in this work were calculated by using both force components. In rare scenarios where one force component showed significant image tracking errors, the other force component was used to calculate the force along the tether.

9. Elastic properties of DNA and theoretical predictions for stretching and unzipping of DNA

dsDNA and ssDNA parameters are both calibrated in a conventional optical tweezers setup in a calibration buffer (15.5 mM Tris-HCl pH 7.8, 0.2 mg/mL BSA, 0.5 mM EDTA, 2.5 mM NaCl, 0.77 mM NaN_3), which is nearly identical to the buffer used for experiments on the nSWAT.

The dsDNA parameters were calibrated by stretching 11.5 kb dsDNA, and the ssDNA parameters were calibrated by unzipping a DNA template with 2.2 kbp arms (total) and 4.1 kbp unzipping segment with DNA hairpin end-capping. dsDNA persistence length and dsDNA elastic modulus are obtained by fitting a modified Marko-Siggia model to measured force-extension curves of 11.5 kb dsDNA molecules using a nonlinear least-squares method[6]. ssDNA persistence length and ssDNA elastic modulus are obtained by fitting to the force-extension curve section after dsDNA is fulling unzipped, with the dsDNA extension portion calculated by the modified Marko-Siggia model using previously determined dsDNA parameters and the ssDNA extension portion calculated by a freely-joined-chain model[7, 8]. The calibrated parameters were: dsDNA persistence length 56.0 $\pm$ 0.7 nm, dsDNA elastic modulus 1199.3 $\pm$ 26.2 pN, ssDNA persistence length 0.9 $\pm$ 0.01 nm, and ssDNA elastic modulus 669 $\pm$ 14 pN.

For comparison, we also directly fit the force-extension curves of single dsDNA tethers shown in Fig. 3c. These fits yielded a persistence length 51.0 $\pm$ 1.8 nm and a stretch modulus 1085.0 $\pm$ 83.2 pN, which are in a reasonably good agreement with those calibrated above.

These calibrated parameters were used to generate the expected theory curves and calculate the number of base pairs of unzipped DNA (Figs. 3, 4 & 5). The theoretical prediction of dsDNA stretching was calculated using these parameters and the modified Marko-Siggia model. The unzipping theoretical prediction was calculated using an equilibrium statistical mechanical

model, which uses a partition function to calculate the expected force and expected number of base pairs unzipped at a given DNA extension[8, 9, 10]. For this calculation, we used the calibrated dsDNA and ssDNA parameters, the known DNA sequence of the unzipping segment, and an estimated temperature of 25 ^{o}C at the specimen plane.

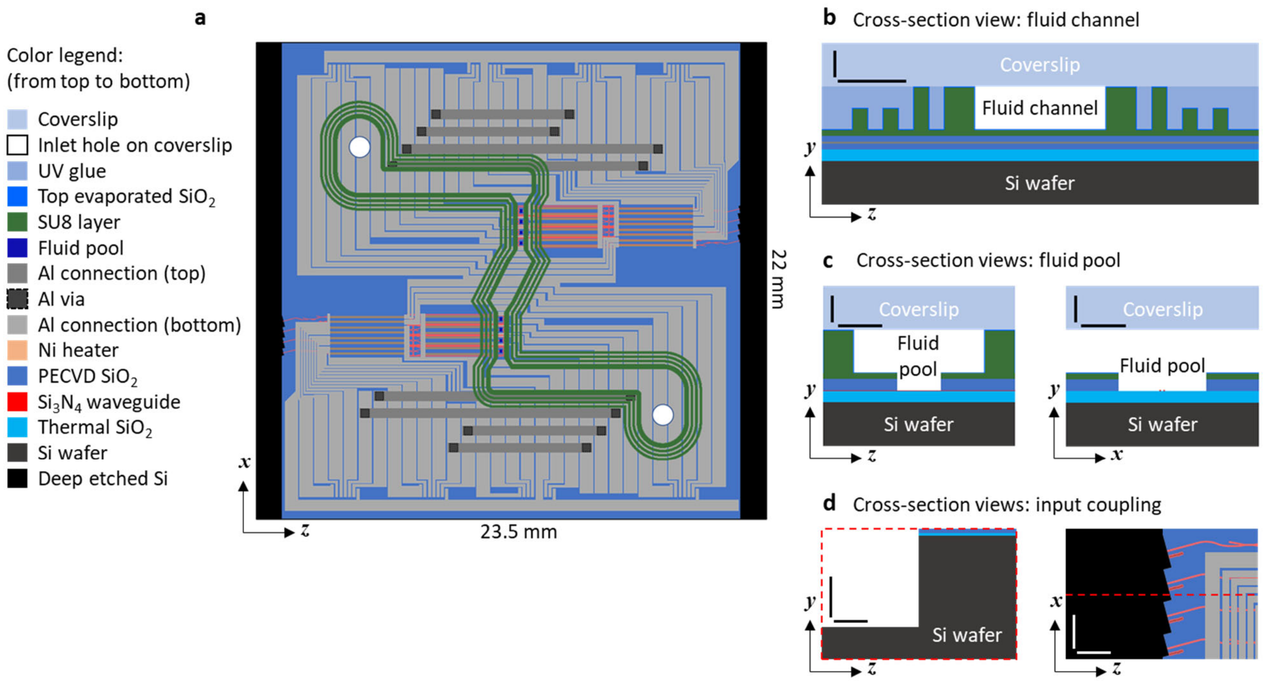

Supplementary Figure 1. Design layout of a resonator-nSWAT chip.

a, Detailed layout of a resonator-nSWAT chip. The fabrication of resonator-nSWAT devices entails a multi-layer process. Each chip contains 8 devices, designed with an inversion symmetry about the center point, such that light is coupled to 4 devices from the left-hand side of the chip and to the remaining 4 from the right-hand side. To better illustrate the design details, several layers of SiO_2, the top SU8 protection layer, and the microscope coverslip are not shown in **a**.

b, Cross-section view of a center horizontal line in **a**. Fluid channel is formed by the coverslip on the top, the nSWAT chip on the bottom, and the two SU8 sidewalls that are fabricated on the chip. The application of a UV glue (see Methods) seals the channel. Scale bars are 500 µm along z and 10 µm along y.

c, Two cross-section views of the fluid pool region. Scale bars are 500 µm along z and along x, and 10 µm along y.

d, Two cross-section views of the input coupling region. The Si wafer is deep etched by 120 µm to facilitate fiber-optic light coupling to an input tapered waveguide on-chip. To minimize in-plane light scattered from the coupling region to the trapping region, the input waveguide is oriented at 15° off the z direction. Scale bars are 500 µm along z and 50 µm along y for the left panel and 500 µm along z and 500 µm along x for the right panel.

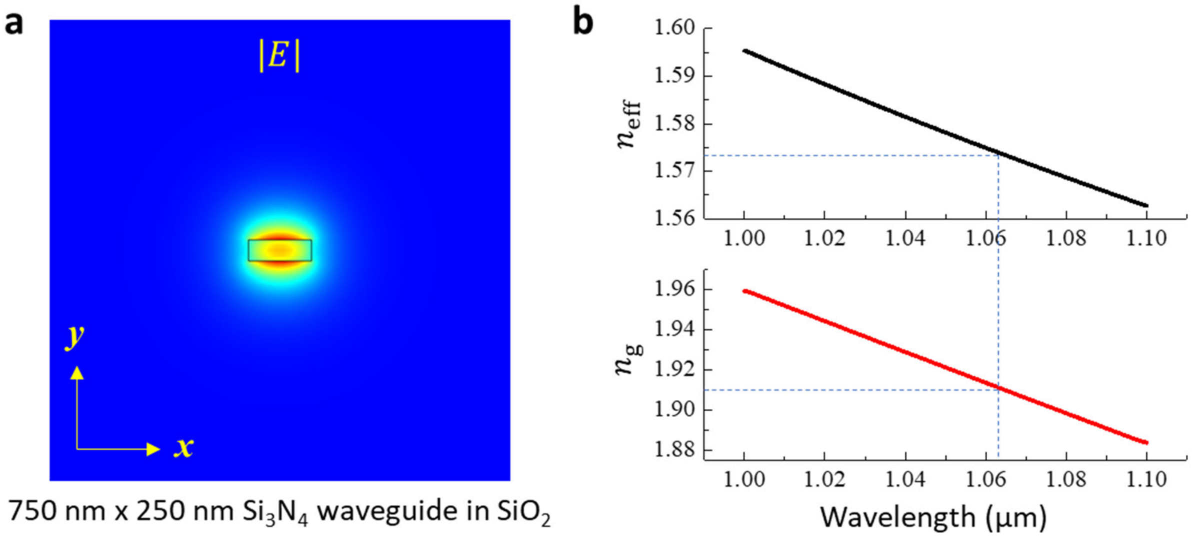

Supplementary Figure 2. Mode profile and dispersion simulation of Si_3N_4 waveguide.

a, TM-like mode profile ($|E|$) in the 750 nm x 250 nm Si_3N_4 waveguide embedded in SiO_2.

b, Simulated effective index of refraction and group index of refraction of the TM-like mode shown in **a**.

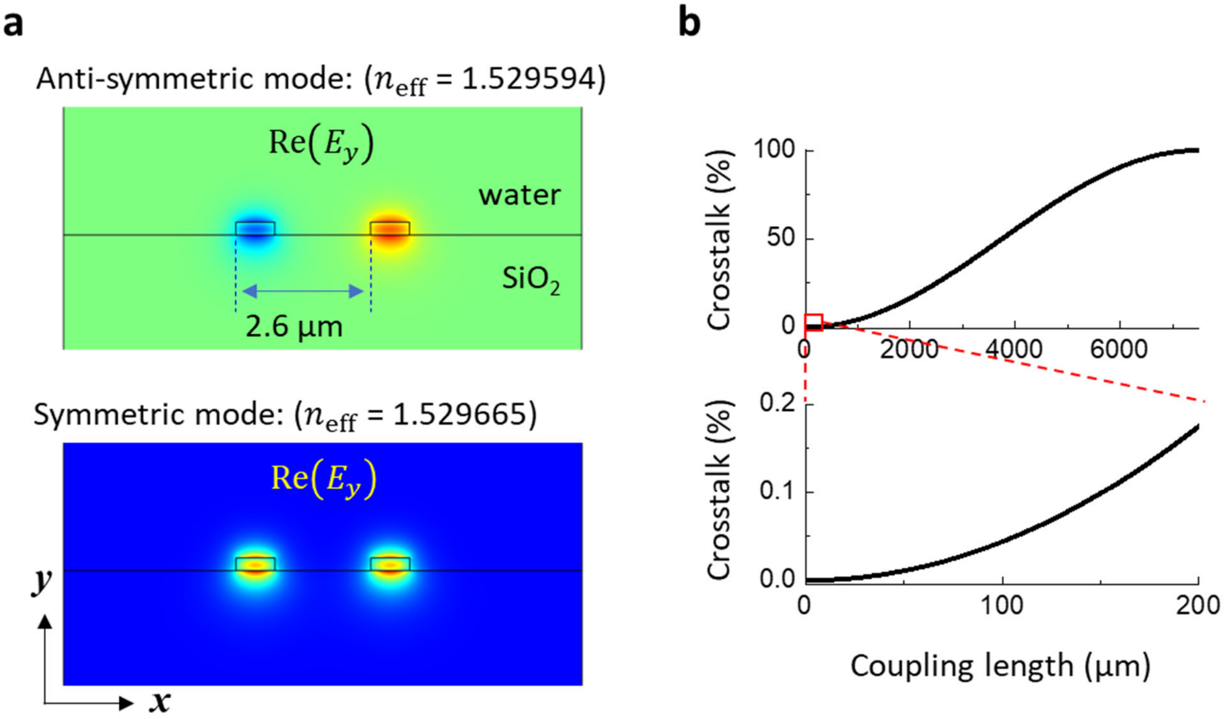

Supplementary Figure 3. Crosstalk estimation between two copies of nSWAT devices.

a, Symmetric and anti-symmetric TM-like modes of the two parallel copies of nSWAT in the fluid pool region. Real parts of E_y are plotted. The difference between the effective indices of these two modes is Δn_{eff} = 0.000071.

b, Percent of the light energy coupled from one copy of nSWAT to the other versus coupling length. At 110 µm coupling length (the length of the fluid pool region where two nSWATs couple) the energy crosstalk is only 0.05%.

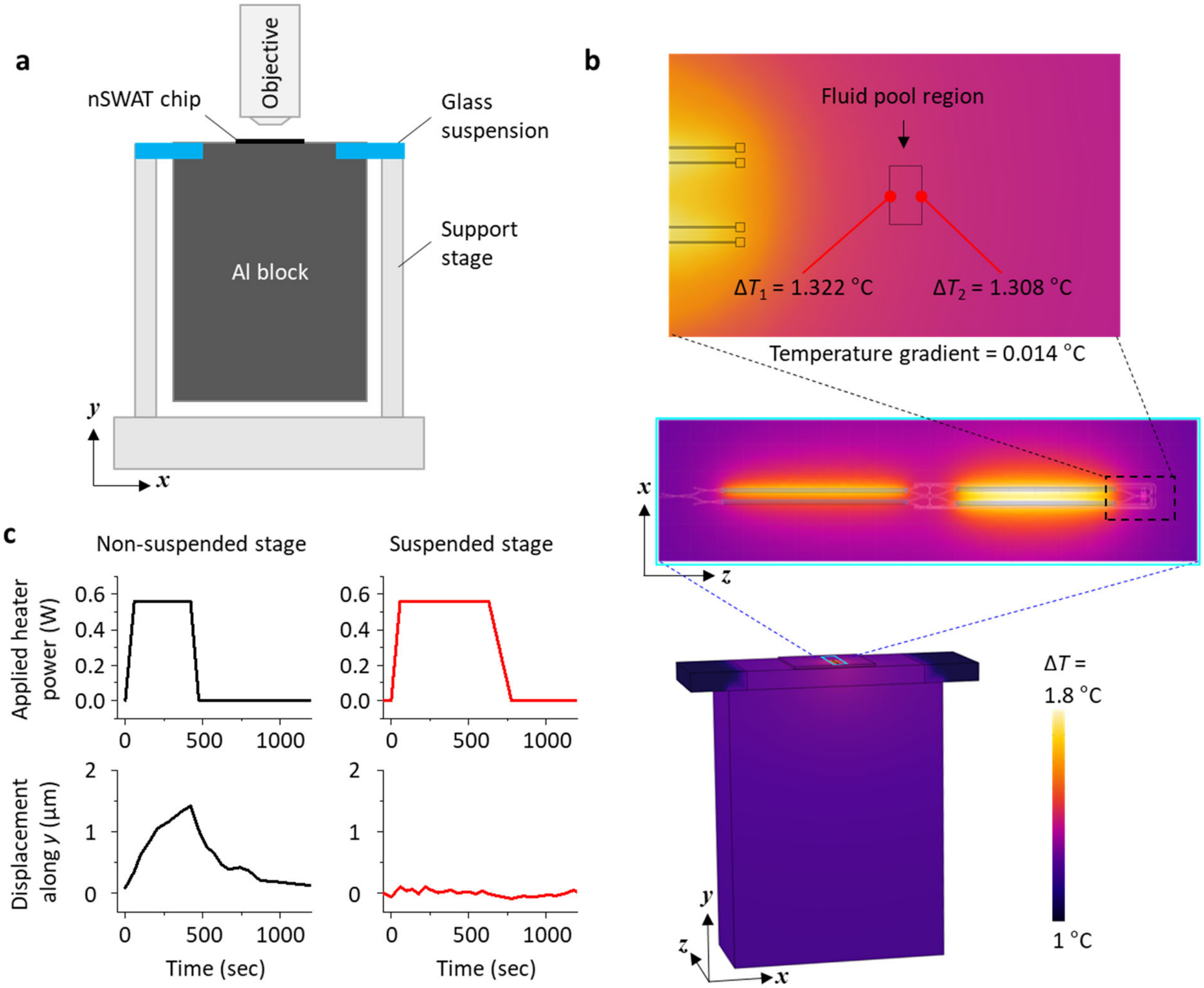

Supplementary Figure 4. Suspended sample stage for minimum thermal drift.

a, Schematic of the suspended sample stage for an nSWAT chip.

b, Temperature increase (ΔT) of an nSWAT chip from COMSOL simulation. The simulation was performed by assuming a constant 0.42 W of heat generated by the microheaters, a condition that resembles those used for a typical experiment. Only the nSWAT chip, the Al block, and the two glass connections were included in the simulation. A convective heat flux boundary condition with heat transfer coefficient of 24 W/(m^{2}K) was applied to all outer surfaces, and the environment was set at 23 ^{o}C air.

c, Measured thermal drift of an nSWAT device during microheater modulation. The thermal drift was measured along the y direction (vertical drift) via maximizing the on-chip IR scattering of the incident laser by tuning a home-built piezo stage. For comparison, we show measurements from both a non-suspended (black) and the suspended (red) sample stage. The non-suspended sample stage configuration directly attaches the bottom of the aluminum block onto the support stage.

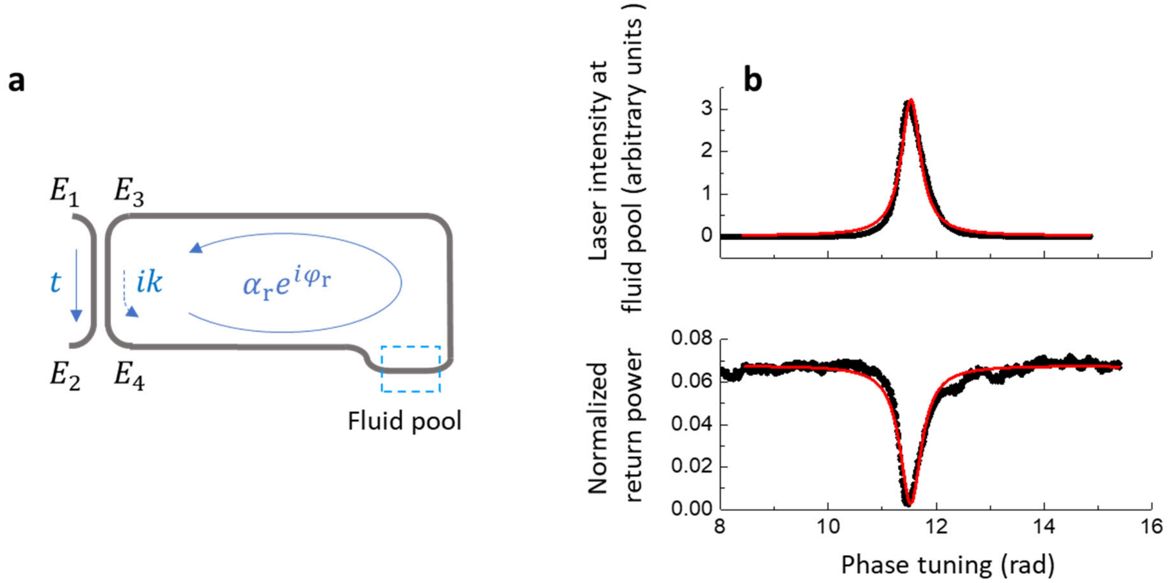

Supplementary Figure 5. Coupling coefficients of the resonator loop.

a, A schematic of the resonator loop illustrating the notation used for the analysis of the coupling coefficients.

b, Resonance curves of local laser intensity and the return laser power and their fits at 0.06 W of input laser power.

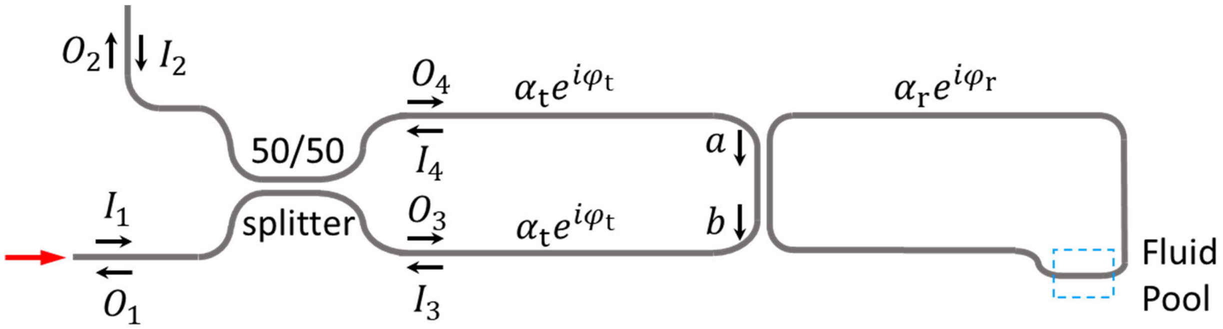

Supplementary Figure 6. Return laser power. This schematic explains the notation used under Methods for the analysis of the return laser power. Here, only a single copy resonator-nSWAT is shown. Light is coupled to the device through port 1. For a perfect 50/50 splitter, light will be eventually returned to port 1 without any light exiting port 2. However, for an imbalanced 50/50 splitter, some light also exits port 2. Under Supplementary Methods, we show that the return laser light to port 1 may be monitored by the light output at port 2.

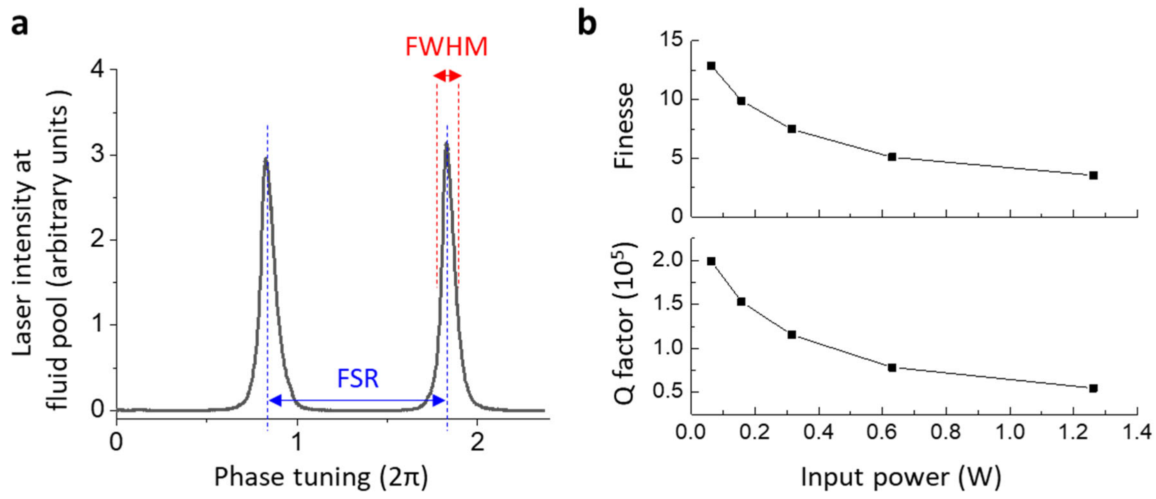

Supplementary Figure 7. Finesse and Q factor determination of the resonator.

a, Laser intensity at the fluid pool region versus phase tuning via microheater. This plot was measured at an input laser power of 0.06 W. Note that a portion of this plot is also shown in Fig. 2.

b, Finesse and Q factor versus input laser power.

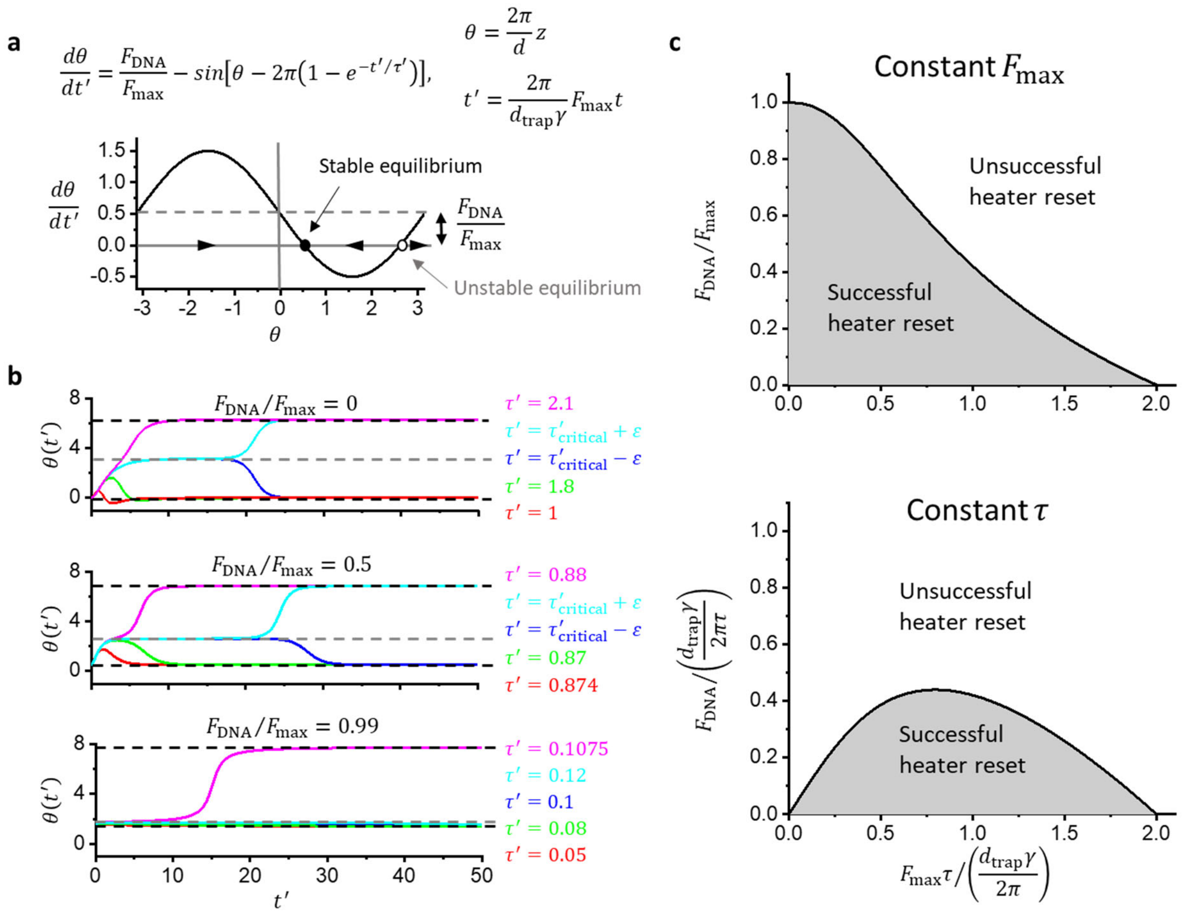

Supplementary Figure 8. Rapid microheater reset allows for an increased manipulation force.

a, Simplified and normalized equation of motion during trap reset. To achieve long-distance motion of the trapped beads, the microheater is reset rapidly after each slow translation of a trap by the trapping potential periodicity, d_{trap}. F_{max} is the maximum force attainable from nSWAT, proportional to the laser intensity in the waveguide. The trap must be reset fast enough so that the bead remains quasi-stationary during the reset, even under the influence of a force from a DNA molecule attached to the bead F_{DNA}. To simplify the equation of motion, F_{DNA} is approximated as a constant for small bead displacements during the trap reset. The position of the trap after the onset of the reset follows an exponential decay with a time constant τ, with $\tau' \equiv \frac{2\pi F_{max}}{d_{trap}\gamma}\tau$ being the normalized time constant and γ being the viscous drag coefficient of the bead. The bead velocity versus bead position in the absence of a trap reset is shown. There is a single stable equilibrium position and an unstable equilibrium position. An increase in F_{DNA} shifts the curve up, bringing the two equilibrium positions closer together until the disappearance of the stable equilibrium.

b, Example bead trajectories. The equation of motion was solved with the MATLAB ode45 numerical solver. For each $\frac{F_{\mathrm{DNA}}}{F_{\mathrm{max}}}$, the τ' at which the bead just arrives at the unstable equilibrium position, $\tau'_{critical}$ is determined. For values of τ'< $\tau'_{critical}$, the nSWAT array moves fast enough that the bead remains quasi-stationary, resulting in a successful trap reset, while for τ'> $\tau'_{critical}$, the bead follows the standing wave array trap during the reset, corresponding to an unsuccessful reset.

c, Maximum force that can be achieved during trap reset. A rapid trap reset time increases the achievable force for stretching DNA F_{DNA}. These curves provide the maximum force from DNA beyond which a bead can no longer be stably trapped. Therefore a fast trap reset time τ is critical to achieving a high force during nSWAT long-range transport. (Top) At a constant F_{max} (constant laser intensity), as the trap reset time τ increases, attainable F_{DNA} decreases monotonically to zero at $\tau = \frac{2}{F_{\mathrm{max}}}\left(\frac{d_{\mathrm{trap}}\gamma}{2\pi}\right)$. (Bottom) At a constant trap reset time τ, the maximum achievable F_{DNA} does not increase monotonically with F_{max} or laser power.

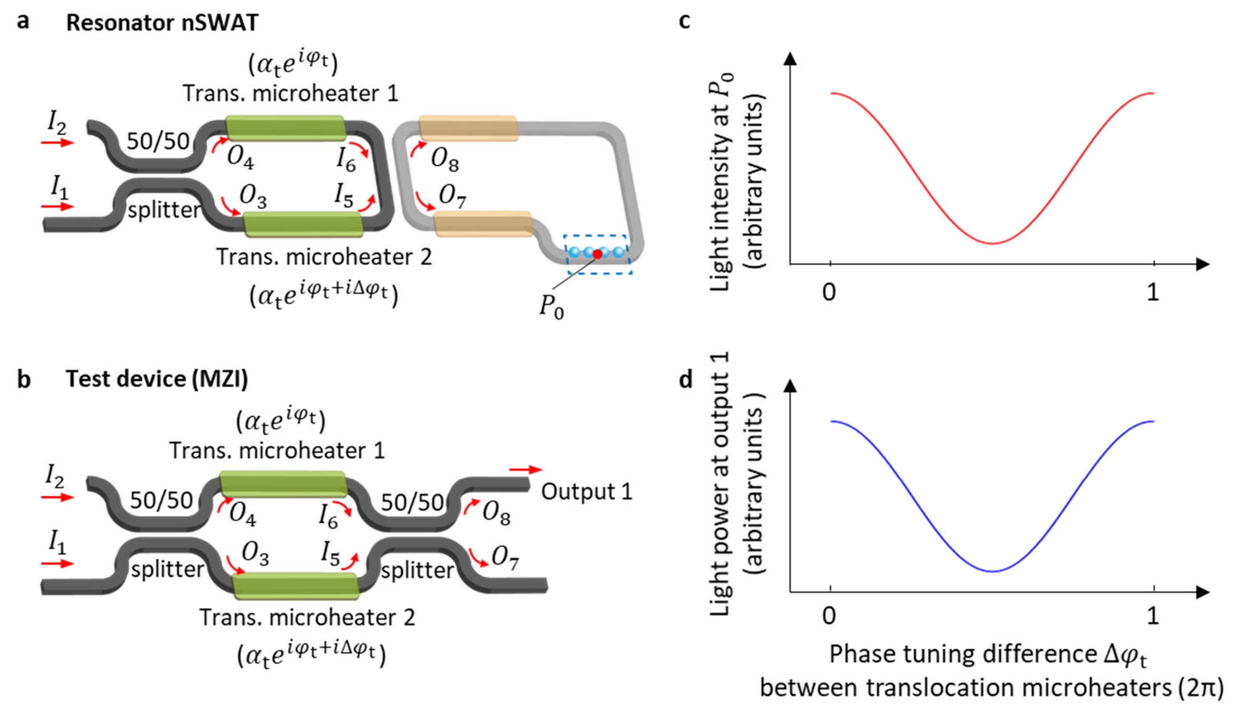

Supplementary Figure 9. Test device for the reset speed of the translocation microheaters.

a, Schematic of a resonator-nSWAT device.

b, Schematic of a MZI test device for the reset speed of the translocation microheaters.

c, Light intensity at position P_0 in the fluid pool of a resonator-nSWAT versus microheater phase tuning on $\Delta\varphi_t$.

d, Output light power at port “output 1” of the test device versus microheater phase tuning on $\Delta\varphi_t$.

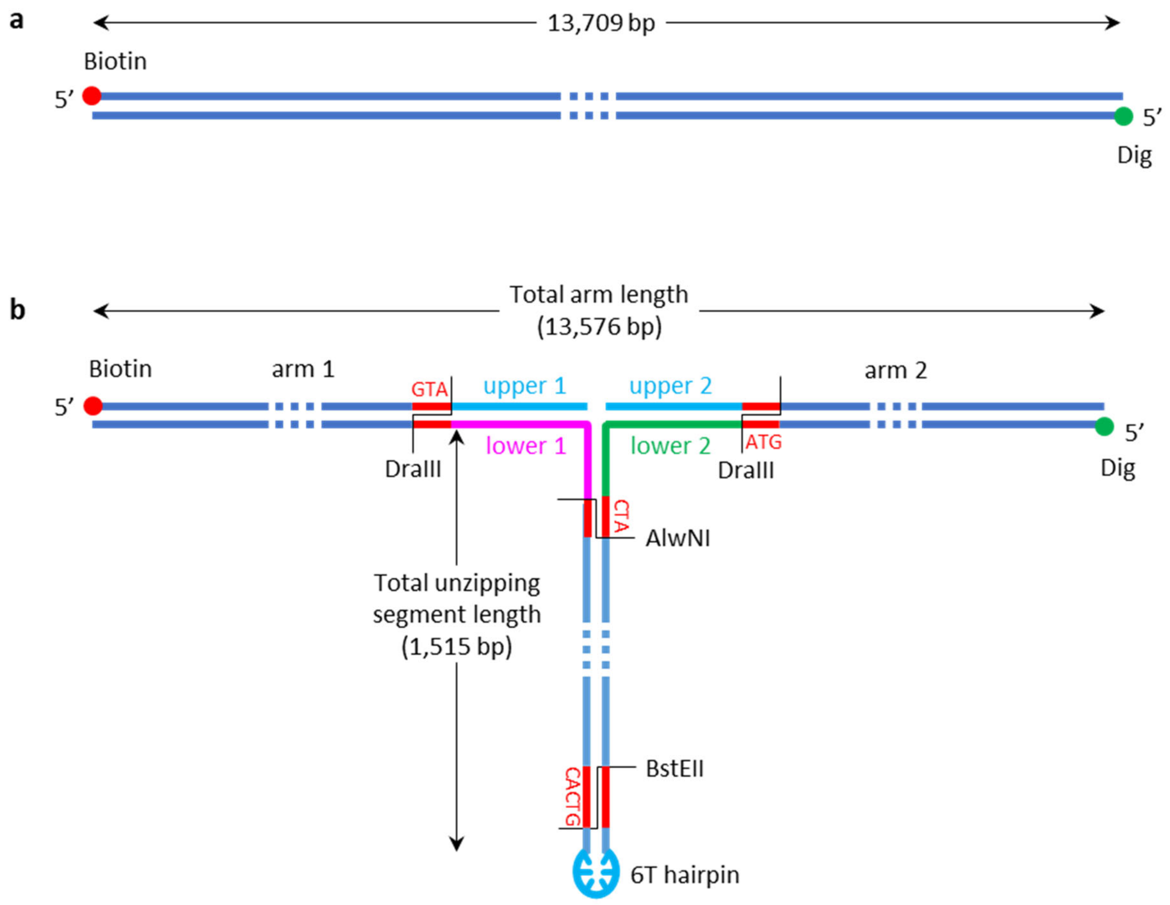

Supplementary Figure 10. DNA template designs.

a, Stretching template design. The dsDNA stretching is 13.7 kbp, with one end labeled by biotin (red dot) and the other end labeled with digoxigenin (dig) (green dot) for attachment to a streptavidin-coated and an anti-dig coated polystyrene bead, respectively (Methods).

b, Unzipping template design. The unzipping template contains two 6.8 kbp dsDNA arms (arm 1 & arm 2) as the unzipping handles and a 1.5 kbp dsDNA unzipping segment capped with a 6T hairpin. These three DNA molecules are ligated to an adaptor DNA formed by annealing 4 pieces of ssDNA molecules (upper 1, lower 1, upper 2, and lower 2).

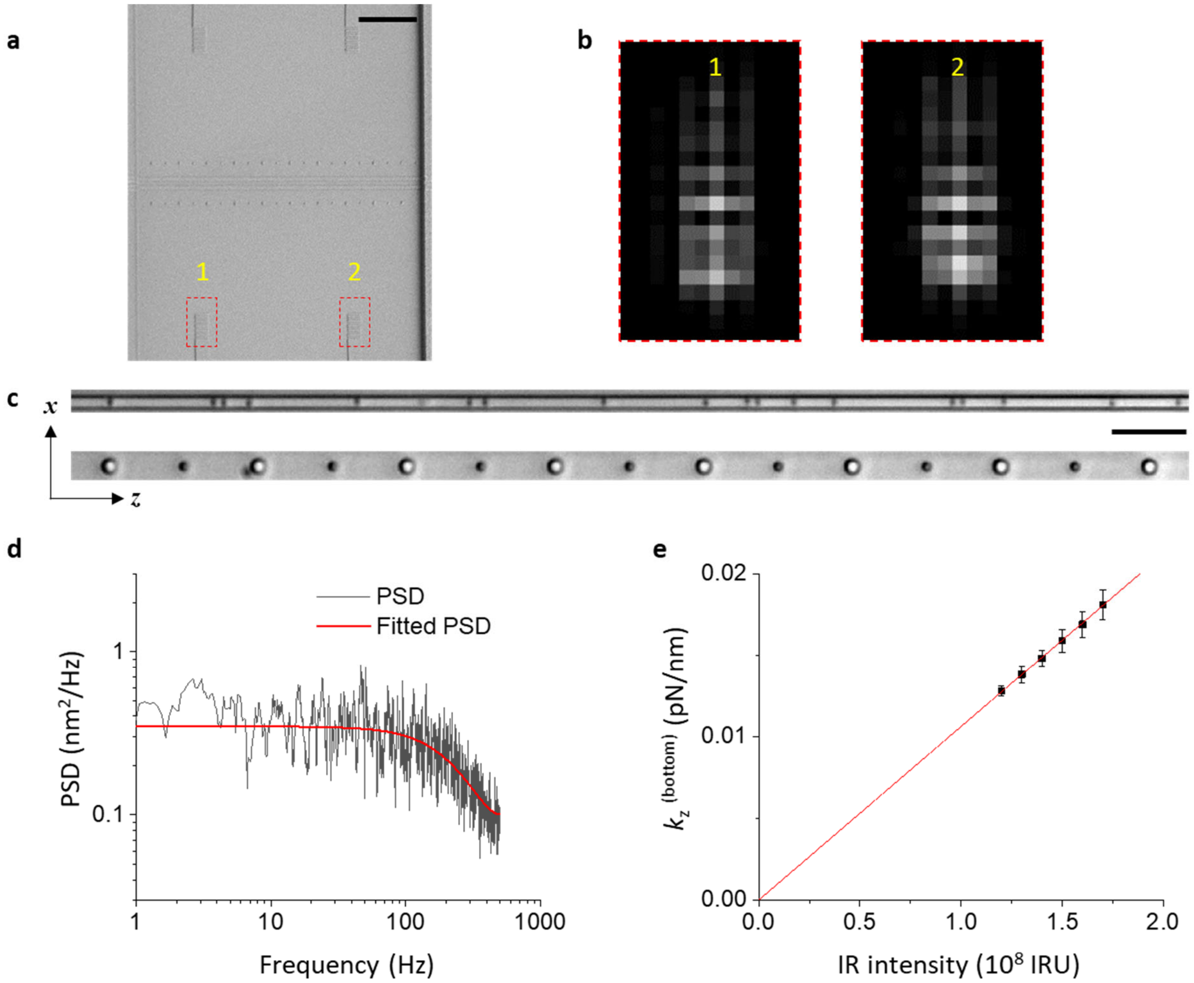

Supplementary Figure 11. Stiffness calibration of a resonator-nSWAT device.

a, SEM image of the fluid pool region of a resonator-nSWAT device. The two scattering centers for the local laser intensity of the bottom waveguide is indicated by red dashed lines and labeled as 1 and 2, respectively. Scale bar: 20 μm.

b, IR camera image of scattering regions 1 and 2 labeled in a, respectively. The average of the sum of the IR intensity in both regions of the image is used to indicate the IR intensity of the nSWAT.

c, Cropped bright field image (under blue LED illumination) of the bottom nSWAT waveguide and the bottom fiducial markers. During trap stiffness calibration, motions of trapped 380 nm beads (dark dots along the waveguide) are recorded and tracked to obtain trap stiffness. Scale bar: 10 μm.

d, Power spectrum density of a trapped bead position along z at local laser intensity = 1.4×10^8 IR intensity unit (IRU). The spectrum was fit by a modified Lorentzian function after corrections of camera blurring and aliasing effects (for details, see Methods section). The fit yielded the corner frequency f_c 273 Hz, the viscous drag coefficient 8.7×10^{-6} pN sec/nm, and the inherent position detection error ε^2 25 nm^2. Thus, the trap stiffness k_z is 0.015 pN/nm.

e, Measured stiffness along z versus local IR intensity. Each data point represents the mean stiffness measured from multiple beads trapped on the nSWAT (*N* = 12). The error bars indicate SEMs. The fitted slope is $(1.06 \pm 0.01) \times 10^{-10}$ pN/nm/IRU. This stiffness calibration was performed for each device that was used for force measurements. Over 30 devices were calibrated, and their calibrations had similar results.

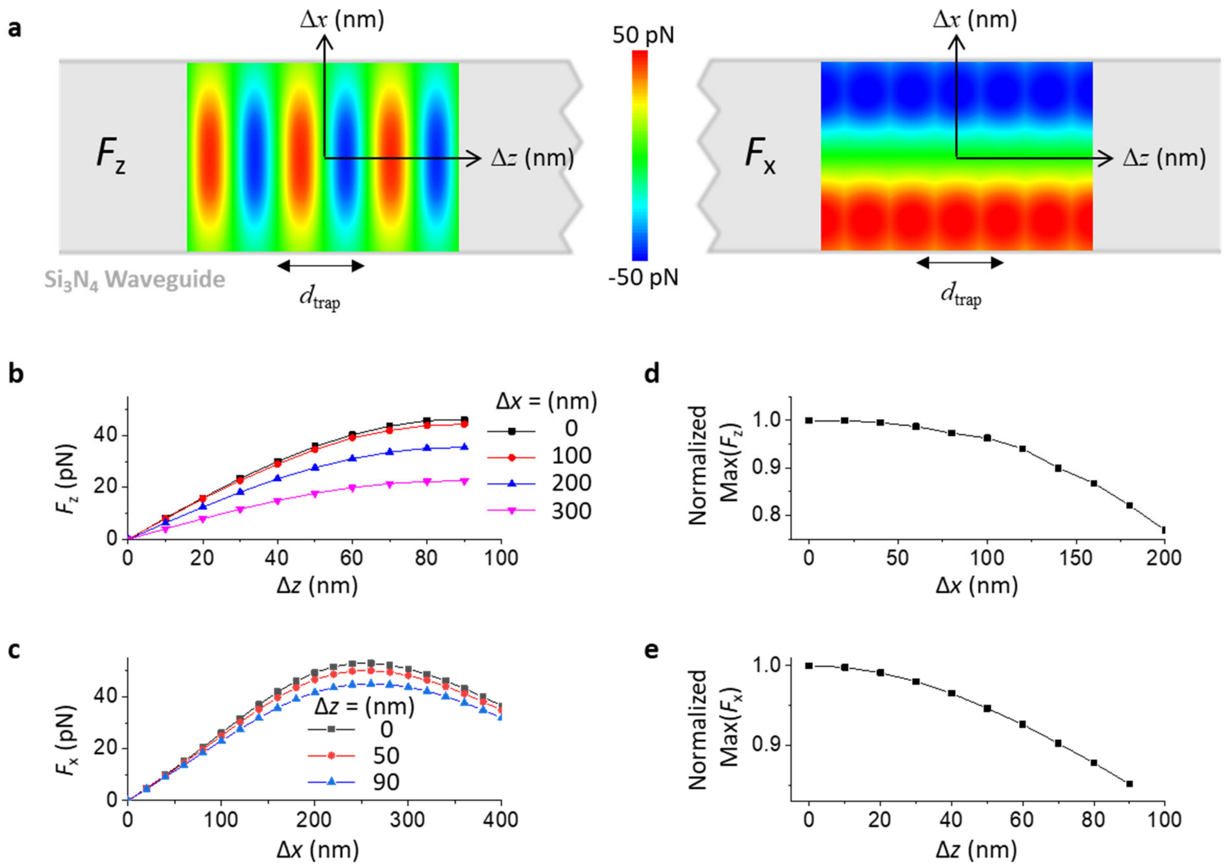

Supplementary Figure 12. 2D trapping force analysis based on simulation.

a, 2D contour map of trapping forces along x and z based on COMSOL simulation. The simulation was performed for a 380 nm polystyrene microsphere at 5 nm above the Si_3N_4 waveguide (cross-section 250 nm x 750 nm) as a function of x and z displacement from the trap center at a total of 1 W power at the trapping region. Simulated trap period d_{trap} is 360 nm.

b, Trapping force magnitude along z versus Δz when keeping offsets along x at different fixed values.

c, Trapping force magnitude along x versus Δx when keeping offsets along z at different fixed values.

d, Maximum trapping force along z normalized to $\text{Max}(F_z)$ at $\Delta x = 0$ vs. Δx.

e, Maximum trapping force along x normalized to $\text{Max}(F_x)$ at $\Delta z = 0$ vs. Δz.

References:

1. Luke K, Okawachi Y, Lamont MRE, Gaeta AL, Lipson M. Broadband mid-infrared frequency comb generation in a Si3N4 microresonator. *Opt Lett* **40**, 4823-4826 (2015).

2. Gao LH, Lemarchand F, Lequime M. Exploitation of multiple incidences spectrometric measurements for thin film reverse engineering. *Opt Express* **20**, 15734-15751 (2012).

3. Kedenburg S, Vieweg M, Gissibl T, Giessen H. Linear refractive index and absorption measurements of nonlinear optical liquids in the visible and near-infrared spectral region. *Opt Mater Express* **2**, 1588-1611 (2012).

4. Bogaerts W*, et al.* Silicon microring resonators. *Laser Photonics Rev* **6**, 47-73 (2012).

5. Badman RP, Ye F, Caravan W, Wang MD. High Trap Stiffness Microcylinders for Nanophotonic Trapping. *ACS Appl Mater Interfaces* **11**, 25074-25080 (2019).

6. Wang MD, Yin H, Landick R, Gelles J, Block SM. Stretching DNA with optical tweezers. *Biophys J* **72**, 1335-1346 (1997).

7. Hall MA, Shundrovsky A, Bai L, Fulbright RM, Lis JT, Wang MD. High-resolution dynamic mapping of histone-DNA interactions in a nucleosome. *Nature Structural & Molecular Biology* **16**, 124-129 (2009).

8. Killian JL, Inman JT, Wang MD. High-Performance Image-Based Measurements of Biological Forces and Interactions in a Dual Optical Trap. *ACS Nano*, (2018).

9. Koch SJ, Shundrovsky A, Jantzen BC, Wang MD. Probing protein-DNA interactions by unzipping a single DNA double helix. *Biophys J* **83**, 1098-1105 (2002).

10. Inman JT*, et al.* DNA y structure: a versatile, multidimensional single molecule assay. *Nano Lett* **14**, 6475-6480 (2014).
