# Supplementary material for: Resonator nanophotonic standing-wave array trap for single-molecule manipulation and measurement
Source: Nat Commun. 2022 Jan 10;13:77. doi: 10.1038/s41467-021-27709-3 (PMC8748738; doi:10.1038/s41467-021-27709-3)
Supplement: Supplementary file 2 — Description of Additional Supplementary Files [file 41467_2021_27709_MOESM2_ESM.docx]

**Description of Additional Supplementary Files**

**Supplementary Movie 1. Principle of an nSWAT device.**

This animation illustrates the design principle of an nSWAT device. Here, an incident laser in a nanophotonic waveguide passes through a 50/50 beam splitter, resulting in counter-propagating waves that form a standing wave. At the antinodes of the evanescent field of the standing wave are an array of trap centers for trapping particles. To translocate the array trap, “translocation microheaters” located over the waveguide modulate the phase of the light via the thermo-optic effect and, therefore, the position of the standing wave.

**Supplementary Movie 2. Simulation of standing wave formation in an nSWAT loop.**

This animation from COMSOL simulation illustrates the process of standing wave formation in an nSWAT loop. A TM-mode laser light along a Si_3_N_4_ waveguide (region in between two black lines) is introduced to an nSWAT loop at time 0. After encountering the 50/50 splitter, the beam is split into two counter-propagating waves, which meet at the center of the nSWAT loop at 130 femtoseconds (fs) to form a standing wave. The standing wave populates the whole nSWAT loop within 100 fs. Due to reciprocity, all incident laser is fully returned to the incident waveguide, forming a standing wave with twice the intensity inside the nSWAT loop. Instant light intensity (|*E*|^2^) is shown in this COMSOL simulated animation.

**Supplementary Movie 3. Simulation of standing wave translocation in a resonator-nSWAT.**

This animation from COMSOL simulation illustrates the control of standing wave positions inside the resonator by modulating the phase difference between two translocation microheaters in the bus waveguide. The incident laser light forms a standing wave in the bus waveguide loop and subsequently in the resonator. To translocate the standing wave in the resonator, translocation microheaters in bus waveguide are used to generate a phase difference (*Δ*phase) between the two counter-propagating waves in the bus waveguide loop. This *Δ*phase also leads to standing wave translocation in the bus waveguide loop, which further leads to standing wave translocation inside the resonator. The video shows the time-averaged light intensity <|*E*|^2^>.
