# Supplementary material for: Resonator nanophotonic standing-wave array trap for single-molecule manipulation and measurement
Source: Nat Commun. 2022 Jan 10;13:77. doi: 10.1038/s41467-021-27709-3 (PMC8748738; doi:10.1038/s41467-021-27709-3)
Supplement: Supplementary file 6 — Reporting Summary [file 41467_2021_27709_MOESM6_ESM.pdf]

nature portfolio

Corresponding author(s): Michelle D. Wang

Last updated by author(s): Nov 19, 2021

Reporting Summary

Nature Portfolio wishes to improve the reproducibility of the work that we publish. This form provides structure for consistency and transparency in reporting. For further information on Nature Portfolio policies, see our Editorial Policies and the Editorial Policy Checklist.

Statistics

For all statistical analyses, confirm that the following items are present in the figure legend, table legend, main text, or Methods section.

n/a	Confirmed	
☐	☒	The exact sample size (*n*) for each experimental group/condition, given as a discrete number and unit of measurement
☐	☒	A statement on whether measurements were taken from distinct samples or whether the same sample was measured repeatedly
☒	☐	The statistical test(s) used AND whether they are one- or two-sided<br>*Only common tests should be described solely by name; describe more complex techniques in the Methods section.*
☒	☐	A description of all covariates tested
☒	☐	A description of any assumptions or corrections, such as tests of normality and adjustment for multiple comparisons
☐	☒	A full description of the statistical parameters including central tendency (e.g. means) or other basic estimates (e.g. regression coefficient) AND variation (e.g. standard deviation) or associated estimates of uncertainty (e.g. confidence intervals)
☒	☐	For null hypothesis testing, the test statistic (e.g. *F*, *t*, *r*) with confidence intervals, effect sizes, degrees of freedom and *P* value noted<br>*Give P values as exact values whenever suitable.*
☒	☐	For Bayesian analysis, information on the choice of priors and Markov chain Monte Carlo settings
☒	☐	For hierarchical and complex designs, identification of the appropriate level for tests and full reporting of outcomes
☒	☐	Estimates of effect sizes (e.g. Cohen's *d*, Pearson's *r*), indicating how they were calculated

Our web collection on statistics for biologists contains articles on many of the points above.

Software and code

Policy information about availability of computer code

Data collection	LabVIEW 2017 nSWAT data collection software
Data analysis	LabVIEW 2020 nSWAT data analysis software

For manuscripts utilizing custom algorithms or software that are central to the research but not yet described in published literature, software must be made available to editors and reviewers. We strongly encourage code deposition in a community repository (e.g. GitHub). See the Nature Portfolio guidelines for submitting code & software for further information.

Data

Policy information about availability of data

All manuscripts must include a data availability statement. This statement should provide the following information, where applicable:

- Accession codes, unique identifiers, or web links for publicly available datasets
- A description of any restrictions on data availability
- For clinical datasets or third party data, please ensure that the statement adheres to our policy

Relevant source data for main text and Supplementary Figures are provided in the Source Data section. All other data that support the findings of this study are available from the corresponding author upon reasonable request.

Field-specific reporting

Please select the one below that is the best fit for your research. If you are not sure, read the appropriate sections before making your selection.

☒ Life sciences ☐ Behavioural & social sciences ☐ Ecological, evolutionary & environmental sciences

For a reference copy of the document with all sections, see nature.com/documents/nr-reporting-summary-flat.pdf

Life sciences study design

All studies must disclose on these points even when the disclosure is negative.

Sample size	The nSWAT can perform multiple, independent, single molecule experiments in parallel. For the data represented here, we collected DNA-tethers on the nSWAT until n ~ 7 for DNA stretching (Figure 1c) and n ~ 4 for DNA unzipping. This sample size was selected to ensure that, after data processing, there were multiple successful traces, thus demonstrating the parallel nature. Each of the parallel experiments represented was replicated in its entirety twice with similar results.
Data exclusions	No data were excluded from the analysis.
Replication	For the DNA-tether experiments (Figures 3-5), we have replicated each experiment at least one time.
Randomization	Randomization does not apply to this type of single-molecule studies.
Blinding	Blinding does not apply to this type of single-molecule studies.

Reporting for specific materials, systems and methods

We require information from authors about some types of materials, experimental systems and methods used in many studies. Here, indicate whether each material, system or method listed is relevant to your study. If you are not sure if a list item applies to your research, read the appropriate section before selecting a response.

Materials & experimental systems

n/a	Involved in the study	
☐	☒	Antibodies
☒	☐	Eukaryotic cell lines
☒	☐	Palaeontology and archaeology
☒	☐	Animals and other organisms
☒	☐	Human research participants
☒	☐	Clinical data
☒	☐	Dual use research of concern

Methods

n/a	Involved in the study	
☒	☐	ChIP-seq
☒	☐	Flow cytometry
☒	☐	MRI-based neuroimaging

Antibodies

Antibodies used	Anti-Digoxigenin from sheep (Sigma, 11333089001 Roche, Lot# 12071900) was used in the study. The Anti-Digoxigenin was dissolved in 0.2 M Borate acid buffer pH 8.5 at the concentration of 2 mg/mL and was then used for coating the carboxylated polystyrene beads.
Validation	Anti-Digoxigenin is a standard reagent for immobilizing the dig-labeled DNA on a surface in single-molecule field. In this work, we covalently coated Anti-Digoxigenin on the carboxylated polystyrene beads to anchor the DNA molecules for single-molecule study. Anti-Digoxigenin from sheep is specific to digoxigenin and digoxin and shows no cross-reactivity with other steroids, such as human estrogens and androgens (https://www.sigmaaldrich.com/US/en/product/roche/11333089001).
